# Engineering phase and polarization singularity sheets

**DOI:** 10.1038/s41467-021-24493-y

**Published:** 2021-07-07

**Authors:** Soon Wei Daniel Lim, Joon-Suh Park, Maryna L. Meretska, Ahmed H. Dorrah, Federico Capasso

**Affiliations:** 1 Harvard John A. Paulson School of Engineering and Applied Sciences, Harvard University, Cambridge, MA USA; 2 Nanophotonics Research Centre, Korea Institute of Science and Technology, Seoul, Republic of Korea

**Keywords:** Metamaterials, Sub-wavelength optics, Optical manipulation and tweezers, Micro-optics

## Abstract

Optical phase singularities are zeros of a scalar light field. The most systematically studied class of singular fields is vortices: beams with helical wavefronts and a linear (1D) singularity along the optical axis. Beyond these common and stable 1D topologies, we show that a broader family of zero-dimensional (point) and two-dimensional (sheet) singularities can be engineered. We realize sheet singularities by maximizing the field phase gradient at the desired positions. These sheets, owning to their precise alignment requirements, would otherwise only be observed in rare scenarios with high symmetry. Furthermore, by applying an analogous procedure to the full vectorial electric field, we can engineer paraxial transverse polarization singularity sheets. As validation, we experimentally realize phase and polarization singularity sheets with heart-shaped cross-sections using metasurfaces. Singularity engineering of the dark enables new degrees of freedom for light-matter interaction and can inspire similar field topologies beyond optics, from electron beams to acoustics.

## Abstract (web summary)

Metasurfaces allow for vast possibilities of light control. Here, the authors demonstrate on-demand engineering and realization of a broad family of two-dimensional phase singularity sheets and transverse polarization singularity sheets, opening up new aspects of light-matter interaction.

## Introduction

Optical phase singularities are zeros in a complex scalar field—regions of darkness surrounded by light^1^. These singularities are ubiquitous in complex wave systems^2^ and typically arise due to rapid or discontinuous phase variation. One common manifestation of this is encountered in helical beams with orbital angular momentum (OAM), which exhibit a linear singularity along the optical axis, around which the wavefront (and thus the Poynting vector) swirls and forms an optical vortex. Phase structures of this nature have attracted much attention owing to the non-intuitive behavior they can imprint upon the surrounding field^3^. For instance, due to ring singularities around the Airy disk in the focal plane of a lens, there are locations near the singularity which have time-averaged Poynting vectors that point back towards the lens, thereby exhibiting locally backward energy flow^4,5^. Furthermore, superoscillations, whereby a bandlimited signal exhibits rapid spatial variation that can be arbitrarily larger than its maximum Fourier component^6^, also typically occur by virtue of nearby singularities^7^. Moreover, phase singularities exhibit different topologies as compared to bright regions and obey different constraints. For example, the diffraction limit constrains the focusing of light. There is no diffraction limit for dark: one can make phase singularities arbitrarily localized and measure their locations with deeply subwavelength precision, limited only by the signal-to-noise ratio of the measuring apparatus^8^. Indeed, measuring an intensity minimum is far more precise than measuring the displacements of finite-width beams of light^9^, an insight that enables a host of applications. Molecular scale imaging enabled via the MINFLUX technique^10^ can infer the position of a fluorescent particle from the spot of minimum emission when the particle is illuminated with a doughnut-shaped pump beam with zero on-axis intensity. Recently, the precise position of the singularities was also exploited to create a subwavelength ruler^8^ that can resolve displacements to better than *λ*/800.

Optical phase singularities such as those along the axis of OAM beams are widely studied one-dimensional (1D) singularity lines. Much is also known about the stochastic properties of 1D singularities in random fields such as laser speckle^11^. However, not all phase singularities are 1D. One-dimensional structures are indeed the most common singular features, as they are stable under small perturbations of the scalar field^12^. Although less common, point (0D) and surface (two-dimensional (2D)) singular structures also exist. Similar to high-order optical vortices, these rare structures are structurally unstable as a small-field perturbation can deform its singular region, removing the singularity altogether, or reducing it to a collection of stable 1D singularities. These singular structures are typically only observed in systems with high symmetry (“degenerate” cases), such as the planar destructive interference fringes in Young’s double slit experiment, as pointed out by Nye and Berry^1^ in their seminal paper predating singular optics, and the radial nodes in cylindrically symmetric diffraction-less Bessel beams^13^.

Here we show on-demand engineering of sheet (2D) singularities in scalar fields without the symmetry constraints. We do so by enforcing large, directed phase gradients at the target singular locations to produce dark sheets. We perform this phase gradient maximization in linearly polarized fields to produce phase singularities and separately in the paraxial vectorial electric field to produce transverse polarization singularities. We validate these numerical predictions experimentally using metasurfaces which implement the required wavefront profile. In contrast to existing studies, our approach does not specify the analytical mathematical description of the field in the vicinity of the singularities^14,15^, as in the construction of a superoscillating function to form an array of optical vortices^16^. Instead, we sculpt singularities based on phase gradients at the position of the singularity itself. This enables the dark regions to be realized with remarkably high contrast and fidelity. Singularity engineering points to the possibility of designing complex beams combining structured light and structured dark, and may inspire exotic field topologies in wave physics beyond optics, from electron beams to acoustics.

## Results

### Singularity geometries and structural stability

**Fig. 1:**
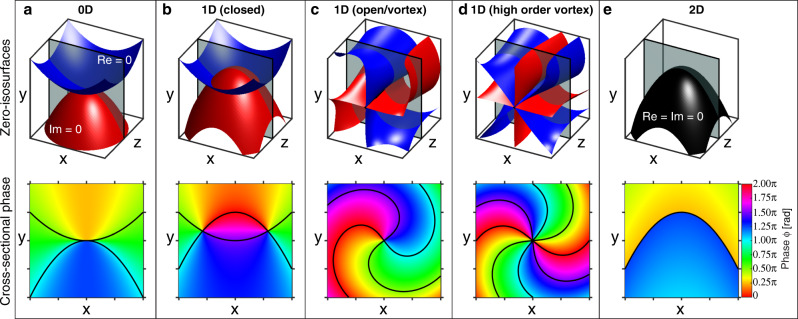
Intersection topologies of zero-isosurfaces that form optical singularities. Optical singularities are formed at the intersection of the zero-isosurfaces of the real (Re) and imaginary (Im) parts of a complex field (e.g., a linearly polarized electric field). Top row: intersection topologies of the two zero-isosurfaces (blue: Re = 0, red: Im = 0). Bottom row: phase profiles for each intersection topology in the top row, evaluated at the gray cross-sectional plane. Black lines indicate the zero-valued contours on the cross-sectional plane. **a** When the zero-isosurfaces intersect at a point, a 0D point singularity is formed. 1D singularities can be formed as **b** a closed loop or **c**, **d** an open line. **c** The fundamental Laguerre–Gaussian_0,1_ vortex beam, with its corkscrew-like zero-isosurfaces. **d** A higher-order Laguerre–Gaussian_0,2_ vortex beam has more than one pair of zero-sheets intersecting along a line. **e** When the zero-isosurfaces coincide, the singularity is 2D.

Singularities refer to undefined parameter(s) of a field. Phase singularities occur where real and imaginary parts of a scalar time-harmonic (with *e*^*−iωt*^ convention here) field *E*(***r***) = Re[*E*(**r**)] + *i*Im[*E*(**r**)] are simultaneously zero, thus leaving the phase *ϕ*(**r**) = arg(*E*(**r**)) undefined there. In most complex three-dimensional (3D) scalar fields, the points at which the real (or imaginary) part of the field vanishes forms a 2D surface: a zero-isosurface. The optical singularities thus occur at the intersection of these two zero-isosurfaces (Re[*E*(**r**)] = 0 and Im[*E*(**r**)] = 0). The intersection may occur at exactly one point where the zero-isosurfaces touch, forming a point singularity (Fig. 1a). By bringing the two zero-isosurfaces closer together, the intersection locus becomes a closed loop (Fig. 1b). Linear singularities can also be open, extending to infinity, as in the widely known fundamental Laguerre–Gaussian_0,1_ (LG_0,1_) vortex beam (Fig. 1c), where the real and imaginary zero-isosurfaces only cross on the optical axis. Cross-sections of these 1D singularities demonstrate that the complex phase swirls in either a clockwise or anticlockwise manner around the singularity locus, accumulating ±*π*/2 over each of the four quadrants defined by the zero-isolines. Higher-order versions of these 1D geometries, such as the second-order LG_0,2_ beam, can be produced if the intersection line coincides with more than one sheet from each of the real and imaginary zero-isosurfaces (Fig. 1d). Notably, when the two zero-isosurfaces coincide, a 2D sheet singularity is produced (Fig. 1e).

First-order 1D singular structures differ from the other topologies in terms of their structural stability. Consider adding a small complex number to each of the scalar fields in Fig. 1. Physically, it can be considered an additional plane wave in the long wavelength limit, which, e.g., may arise from a defect or scatterer in the medium. The addition displaces and distorts the zero-isosurfaces. For 0D and 2D topologies, this shift misaligns the precise orientation of the zero-surfaces, destroying the singularity altogether (when the zero-surfaces do not intersect) or producing 1D singular lines (where the new isosurfaces intersect). On the contrary, the perturbation to the 1D singularity fields with first-order singularities in Fig. 1b or c merely causes the intersection to be displaced in space, preserving the existence of the 1D singularity. Higher-order 1D singularities (as in Fig. 1d) also dissociate into collections of first-order 1D singularities (Fig. 1c) in realistic circumstances under perturbation^17^. In summary, first-order 1D singularities are robust against field perturbations, whereas 0D, higher-order 1D, and 2D singularities are not.

One-dimensional singularities are commonly called topological singularities because they possess an invariant quantity, the topological charge, which is conserved under small-field perturbations and with propagation. For simplicity, we focus here on paraxial fields with a well-defined transverse plane. The topological charge *s* = ∮_*C*_(**∇***ϕ*/2π)·d**r** is computed around an enclosing loop *C* (usually chosen to lie on the transverse plane and defined to be positively oriented in the clockwise direction) so that the singularity line penetrates the interior of *C*. When there are no singularities along *C*, *ϕ* is continuous on the closed loop and must return to its original value modulus 2*π*. Thus, *s* is an integer and can be positive, negative, or zero. This same concept can be extended to 2D singularities by picking *C* large enough to encircle the 2D structure on the transverse plane. Care must be taken, however, when dealing with 2D singularities that have a closed transverse cross-section, as for calculating *s*, *C* must be chosen to have both an inner and outer loop (Supplementary Fig. 1). *s* is conserved under small field perturbations and with wave propagation, provided no singular structures cross *C*, and is equal to the sum of the topological charges of the singular structures penetrating the interior of *C*.

Although optical devices can be designed to shape lighted regions of a field according to one’s specifications through iterative techniques such as computer-generated holography, the Gerchberg–Saxton (GS) algorithm^18,19^ or adjoint optimization^20^, or through non-iterative means such as in freestyle laser traps^21,22^, sculpting the dark is more challenging. Iterative light-shaping numerical techniques rely on the reverse propagation of a desired light-field distribution to provide information on how to improve the design of an optical device in the next iteration. These techniques work well if the desired output optical intensity profile comprises lighted regions but fail if the desired pattern is dark or associated with little optical intensity; if there are singular regions in the desired optical output, reverse propagation of the output from these regions will result in zero information provided to the iterative algorithm. One might argue that these limitations can be bypassed by specifying the way the nearby fields are distributed so that the intensity goes to zero at the desired singularity locations. By doing so, one can then construct the closest wave-equation solution to the predefined phase and amplitude distribution using analytic techniques^23^. However, in specifying one spatial pattern adjacent to the singularity, one will reduce the space of acceptable designs by excluding other optical fields that also contain the desired singularity structure, potentially excluding better-behaved fields which more closely approximate the desired field distribution. In short, one must go beyond the standard techniques of optical engineering to manipulate the dark side of light.

### Singularity shaping strategy

**Fig. 2:**
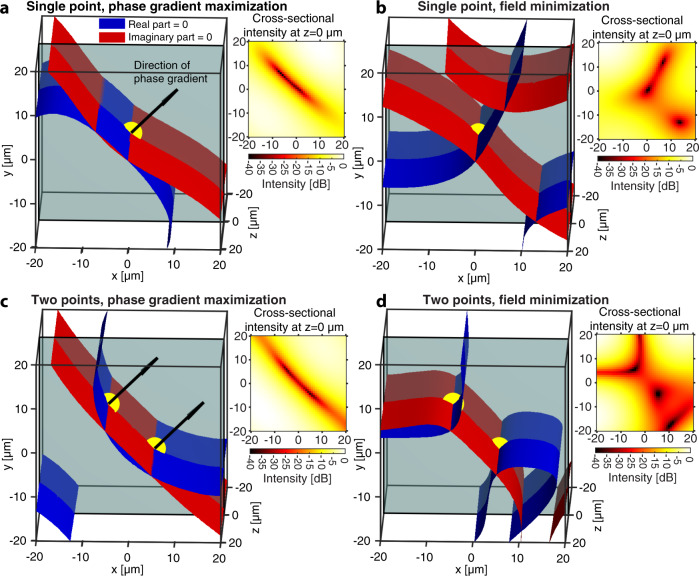
Comparison between phase gradient maximization and field minimization to obtain phase singularities. Both methods can be used to obtain phase singularities, but they produce different field behavior in terms of its real (blue) and imaginary (red) zero-isosurfaces. Yellow dots label the positions at which the field and phase gradients are optimized. Inset surface plots are the logarithmically scaled field intensities at *z* = 0 μm over the same *XY* domain. The *z* = 0 μm plane is indicated with the gray plane in each isosurface plot. **a** When the phase gradient in a specified direction is maximized, the two zero-isosurfaces align approximately tangentially and in the direction normal to that specified gradient. This produces a flat low field intensity structure along these aligned zero-isosurfaces. **b** Minimizing the field amplitude at a point to produce a singularity merely enforces a crossing of the zero-isosurfaces without any alignment, producing a 1D line singularity. **c** Simultaneously optimizing two nearby points with directed phase gradients can extend the range of the singularity sheet. **d** Minimizing the field amplitude at two points simultaneously does not guarantee alignment of the zero-isosurfaces and can instead produce multiple crossing lines, each producing a 1D line singularity.

Phase singularities are intimately connected to phase gradients, which rise to arbitrarily large values in the vicinity of a singularity. Hence, our approach to singularity engineering does not rely on optimizing values of field parameters (e.g., amplitudes or phases), but rather their gradients. An arbitrarily large phase gradient at a point implies that there is vanishing field value there, although the reverse implication is not true (Supplementary Note 1). The magnitude of a phase gradient (normalized to the field wavenumber *k*_0_) is thus a continuous measure of the degree to which a singularity-like optical field approaches true singularity behavior, where an infinite value indicates a mathematical singularity with undefined phase and identically zero amplitude. Unlike the field amplitude, such a measure does not need to be normalized to the overall amplitude scale of the optical field. This connection between phase singularities and phase gradients motivates our approach to inverse-designing the dark. We maximize the spatial derivatives of the phase as a proxy for enforcing phase singular behavior. In particular, as the phase gradient is a vector normal to the wavefront (the surface of constant phase), by maximizing it at a point in a specified direction, we produce an asymptotically zero intensity sheet oriented normal to that direction. Figure 2a demonstrates the result of one such optimization for a single point. A very large and directed phase gradient can be achieved by designing the real and imaginary zero-isosurfaces so that they touch tangentially and are normal to that specified direction, as visualized in Fig. 2a. Notably, minimizing the field amplitude to produce a phase singularity does not produce this alignment; it merely enforces a crossing of the zero-isosurfaces instead (Fig. 2b). The extent of the singularity sheet can be increased by maximizing the phase gradient at multiple nearby positions (Fig. 2c), e.g., by using an objective function that depends on the phase gradient at multiple positions. In contrast, minimizing the field amplitude at two nearby points may not have this alignment effect, often producing multiple discrete intersections instead (Fig. 2d).

Calculating fields with large phase gradients also requires special care in taking the derivative. Finite difference methods should not be used to estimate the phase gradient near singularities where the phase gradients are diverging because it is susceptible to errors, in particular due to the 2*π* phase periodicity. In our approach, we perform all calculations on an algorithmic differentiation (backpropagation) platform. We record the mathematical operations performed in connecting variables (e.g., device design parameters and their positions in space) to their results (e.g., complex field values or objective function values). We then traverse this record using the chain rule for derivatives to obtain the exact numerical derivative of any calculated value with respect to any other variable^24^.

It is important to mention that the 2D singularities we study here may not be absolute mathematical singularities in the sense that the field value along the singularity is small but may not be identically zero. This can be reconciled as follows: first, we chose to explore the parameter space of physically realizable light fields generated through a finite aperture and to find the closest field solutions that maximize the phase gradient locally on a finite set of points. Mathematically exact sheet singularities that are infinitely extended over space typically require an infinite aperture to be replicated perfectly, such as those associated with 1D diffraction patterns^1^ or those similar to Bessel beam nodes^13^ and LG radial nodes^25^. Second, although the phase gradient can become arbitrarily large during optimization, we truncate the optimization process early to avoid dealing with infinities and numerical precision limitations. These approximate singularities may not have zero field value but are close enough to zero with large phase gradients for physically interesting realizations. It is noteworthy that this is also the case for 3D volumetric singularities: although volumetric singularities are not mathematically possible (see Supplementary Note 2), “perfect” optical vortices (i.e., vortices with dark core radii that are both independent of the topological charge and where the largest field gradients are located) with a core volume containing very low light intensity have been designed and generated experimentally^26^.

Although not the focus of this study, we note that one way to deterministically position 0D point singularities is to place them on the axis of a cylindrically symmetric field. Both amplitude minimization and phase gradient maximization can be applied to produce these singularities. Supplementary Fig. 2 plots a simulated cylindrically symmetric field with three deterministically placed 0D singularities. More details are available in the Supplementary Methods.

### Engineered phase-singularity sheet

**Fig. 3:**
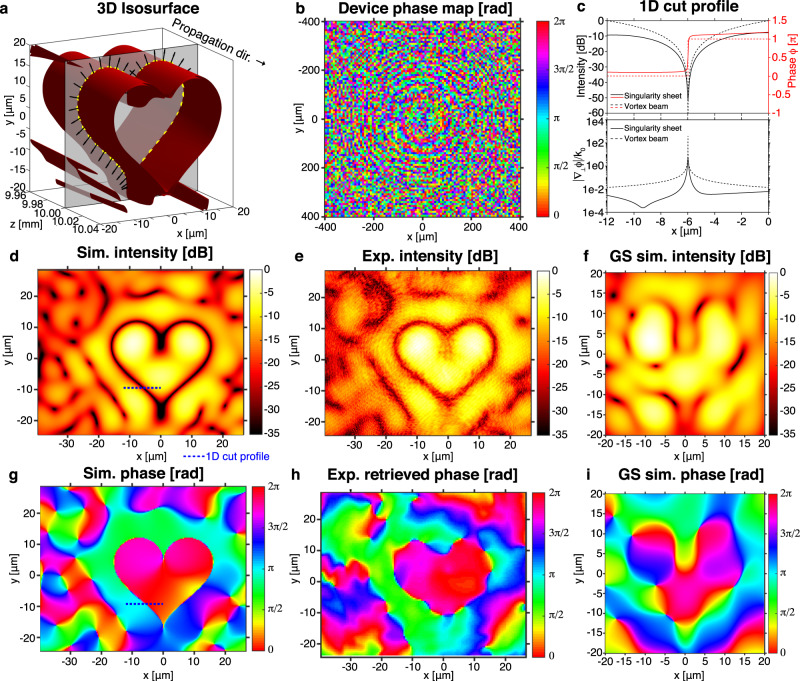
An engineered heart-shaped optical phase-singularity sheet (*λ*_0_ = 532 nm). **a** Isosurface of low field intensity for the simulated singularity sheet. The phase gradient was maximized in the directions indicated by the arrows on the gray *z* = 10 mm plane, at the locations of the yellow dots. **b** Inverse-designed phase profile, located at *z* = 0 mm, which realizes the heart-shaped optical singularity. **c** The 1D cut profile of intensity, phase, and phase gradient magnitude |∇_⊥_*ϕ*| = [(∂_x_*ϕ*)^2^ + (∂_y_*ϕ*)^2^]^1/2^ for the dotted blue line in **d**, overlaid with the corresponding quantities for a Laguerre–Gaussian vortex beam. **d** Simulated relative intensity and **g** phase profile of the singularity sheet, at *z* = 10 mm. **e** Relative intensity profile obtained experimentally with a fabricated metasurface and **h** phase profile (obtained from iterative phase retrieval), at *z* = 10 mm. As a comparison, the results of Gerchberg–Saxton (GS) iterative optimization to get the same heart pattern on the *z* = 10 mm plane are plotted in **f** for the intensity and in **i** for the phase at the target plane, which demonstrates lower pattern fidelity and contrast as compared to the phase gradient maximization result.

As a proof-of-concept for singularity sheet engineering, we design a 2D phase-singularity sheet with a heart-shaped cross-section (Fig. 3). The heart-shaped singularity is centered at *z* = 10 mm and is designed for the scalar field associated with the *x*-polarized electric field at *λ*_*0*_ = 532 nm emitted from a 0.8 mm × 0.8 mm patterned aperture. The paraxial scalar field approximation (qualitatively supported by the propagation distance to the target plane being much larger than the aperture size) is justified by a full vectorial propagation^27^ of the electromagnetic fields, which shows that the time-averaged energy density associated with the *y* (transverse) and *z* (axial) polarization components over the volume of interest is much smaller (in this case, <0.05%) than that of the *x*-polarization. The sheet singularity is constructed by maximizing the phase gradient in the directions oriented normally to a heart-shape at the target *z* = 10 mm plane. The free parameters are the propagation phase delay (from 0 to 2*π*) at each pixel on the patterned aperture located at *z* = 0 mm. The optimized phase pattern is shown in Fig. 3b and does not exhibit any discernible long-scale pattern apart from a series of concentric rings, which appear to apply a focusing effect. Figure 3a shows a field intensity isosurface of the singularity profile to depict its orientation in 3D space (real and imaginary zero-isosurfaces are plotted in Supplementary Fig. 3), along with the locations and directions at which the phase gradient maximization was performed. This surface represents the points at which the phase gradient is very large, nearly reaching 100 times the wavenumber *k*_0_. Simulated cross-sectional intensity and phase plots at the *z* = 10 mm plane are depicted in Fig. 3d, g, respectively. There is a visible phase jump of *π* radians across the singularity boundary as the field changes sign. The phase profile is in fact continuously differentiable with a well-defined phase gradient and just achieves a large gradient value at the singularity boundary. In Fig. 3c, we plot this phase gradient magnitude |∇_⊥_*ϕ*|^2^ = (∂_x_*ϕ)*^*2*^ + (∂_y_*ϕ)*^*2*^ profile alongside the intensity and phase profiles for the linear cut indicated in Fig. 3d. These profiles are qualitatively similar to those of a transverse cut through the singular optical axis of a fundamental LG_0,1_ vortex beam of the same wavelength (with a beam waist diameter equal to the diagonal of the 0.8 mm × 0.8 mm square aperture), which are plotted using dotted lines in Fig. 3c. The transverse phase gradient over the transverse plane is plotted in Supplementary Fig. 4. This phase gradient achieves large magnitudes near the heart-shaped boundary and even exceeds *k*_0_ by an order of magnitude, thereby exhibiting superoscillatory behavior. The longitudinal behavior of this cut profile with *z* is displayed in Supplementary Fig. 5, which shows that |∇_⊥_*ϕ*| exceeds *k*_0_ for a superoscillatory region ~150 μm in front of and behind the target *z* = 10 mm plane. The characteristic depth of this singularity sheet using this superoscillatory region is thus around 300 μm. The depth of a singularity sheet can be extended by maximizing the phase gradient over more than one transverse plane. We also exhibit additional phase-singularity sheet shapes (flat sheet and double-walled cylinders) in Supplementary Fig. 6 to demonstrate the versatility of this algorithm.

Similar to asymmetric optical vortices with fractional topological charge or high-order vortices with topological charge >1, the 2D heart-shaped singularity sheet is unstable with propagation^28,29^ and hence has a finite length in the axial direction. This instability with propagation is not a general characteristic of 2D sheet singularities; Bessel and LG beam nodes and 1D diffraction patterns are notably stable with propagation. The topological charge of our heart singularity sheet measured at the target plane is *s* = 0, computed using the inner and outer curves in Supplementary Fig. 7, indicating that equal numbers of vortices with *s* = +1 and −1 are formed following the break-up of the sheet singularity. The physical interpretation of this quantity is that it is an estimate of the mean OAM per photon *l* associated with a region comprising the singularity, in units of *ħ*. We can explicitly compute *l* over the cross-sectional area *A* between a set of inner and outer curves (Supplementary Fig. 7) using the method proposed by Allen and Padgett^30^, where *l* is given by the ratio of the time-averaged OAM, *J*_*z*_, to the time-averaged energy per unit length, *W* (Eqs. (1–3)).

1 $${J}_{z}={\epsilon_0}\iint_{\!\!\!\!{\rm{A}}}dxdy({\bf{r}}\;\times {\rm{Re}}[{\bf{E}}\;\times\;{\bf{B}}^\ast ]/2)\cdot \hat{z}$$

2 $$W={\rm{c}}{\epsilon_0}{\iint }_{\!\!\!\!{\rm{A}}}dxdy({\rm{Re}}[{\bf{E}}\;\times\;{\bf{B}}^\ast ]/2)\cdot \hat{z}$$

3 $$l={\rm{\omega }}{J}_{z}/W$$

We obtain *l* = −0.0011, which is very close to the topological charge of *s* = 0 obtained by line integration.

### Experimental realization of phase-singularity sheet

**Fig. 4:**
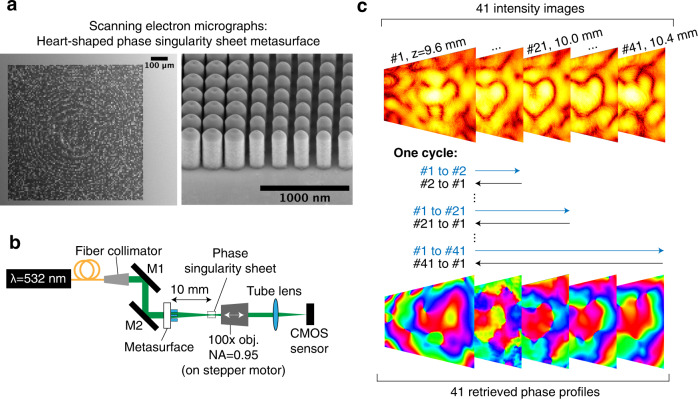
Fabrication and characterization of the heart-shaped phase-singularity sheet. **a** Left, scanning electron micrograph of the fabricated metasurface. This metasurface comprises 101 × 101 superpixels with a pitch of 8 μm. Each superpixel comprises a uniform 32 × 32 array of cylindrical nanopillars with a pitch of 0.25 μm. **a** Right, high-magnification SEM of the interface between two superpixels. **b** Experimental setup for the optical characterization of the heart-shaped phase singularity. The ×100 objective (numerical aperture (NA) = 0.95) is scanned over 41 *z*-positions (from 9.6 to 10.4 mm) to capture the longitudinal variation of the phase singularity using a complementary metal oxide semiconductor (CMOS) camera sensor. **c** Iterative single-beam multiple-intensity reconstruction phase retrieval algorithm used to estimate the phase profile at each *z*-position. During one cycle, the forward propagation is performed from the first image (at *z* = 9.6 mm) to each image and backwards to the first image. This cycle is performed 50 times to yield the 41 phase profiles at each *z*-position.

We validate the numerical predictions experimentally by fabricating a transmissive metasurface to realize the required phase profile in Fig. 3b. Metasurfaces are not the only means to produce sheet singularities; any wavefront shaping device, which can sample the highest spatial frequency of the target pattern, can also be deployed. The metasurface comprises cylindrical nanopillars of TiO_2_ on an SiO_2_ substrate. Scanning electron micrographs of the full metasurface and the nanopillar arrays are exhibited in Fig. 4a, and the experimental setup used to profile the singularity sheet produced is displayed in Fig. 4b. The intensity and reconstructed phase profiles produced by the metasurface at the *z* = 10 mm plane are plotted in Fig. 3e, h, respectively. The phase profile was obtained through single-beam multiple-intensity reconstruction^31^ applied to the measured 3D intensity map, as depicted in Fig. 4c. The intensity and phase profiles show close correspondence to those obtained through simulation in Fig. 3d, g. Furthermore, the rapid intensity decay along the singularity sheet (~ −35 dB), stemming from the phase gradient maximization, can be observed in the plots. Notably, the continuity and uniformity of the dark contour is difficult to achieve using conventional computer-generated holography techniques that rely on amplitude minimization. This can be seen by comparison to Fig. 3f, i, which exhibits the best field intensity and phase obtained by using the GS phase retrieval algorithm to design a similar heart-shaped singular trajectory just on one transverse plane. Supplementary Fig. 8 demonstrates how the GS algorithm is unable to replicate fine intensity features over the transverse plane due to the limited range of transverse spatial frequencies and further details on this can be found in Supplementary Note 3. In addition, Supplementary Fig. 9 displays the cross-sectional intensity and phase profiles of the singularity at various *z*-positions alongside the numerical predictions and are in very good agreement.

### Engineered polarization singularity sheet

The process for engineering 2D phase-singularity sheets is also directly applicable to engineering 2D polarization singularity sheets. A detailed review of the connection between phase and polarization singularities is provided by Ruchi et al.^32^. Polarization singularities can take multiple forms, but here we focus on *C*-point singularities in which the polarization azimuth *Ψ* (in a paraxial field, with negligible *E*_*z*_ contribution) is singular^12,33^. *Ψ* is the angle that the major axis of the polarization ellipse makes with the transverse *x*-direction (Supplementary Fig. 10). Together with the ellipticity angle *θ*, which determines the eccentricity and handedness of the ellipse, the pair (*Ψ*, *θ*) parameterizes the space of fully polarized light. To deploy phase gradient maximization, we need to identify a complex scalar field *σ* such that arg(*σ*) ∝ *Ψ*. This complex field will exhibit the same geometries in Fig. 1. Maximizing the phase gradient of *σ* then maximizes the azimuth gradient and produces polarization singularities. One such field is *σ* = |*E*_*x*_ | ^2^ − |*E*_*y*_ | ^2^ + 2*i*·Re(*E*_*x*_*E*_y_^*^) for which arg(*σ*) = *2Ψ*. This can be written as *σ* = *s*_*1*_ + *is*_*2*_ using the Stokes polarization parameters, which comprise four experimentally measurable intensities that parametrize the full space of polarized (and partially polarized) light. They are defined in Eqs. (4)–(7) using the left-handed convention^34^.

4 $${s}_{{0}} \!\!= \!\!{I}_{x}+{I}_{y}=|{E}_{x}{|}^{2}+|{E}_{y}{|}^{2}$$

5 $${s}_{{1}} \!\!= \!\!{I}_{x}\mbox{-}{I}_{y}=|{E}_{x}{|}^{2}\mbox{-}|{E}_{y}{|}^{2}$$

6 $${s}_{{2}} \!\!= \!\!{I}_{{45}{^\circ }}\mbox{-}{I}_{{-}{45}{^\circ }}=2\cdot {\rm{Re}}({E}_{x}{{E}_{y}}^{\ast })$$

7 $${s}_{{3}} \!\!= \!\!{I}_{{\rm{LCP}}}\mbox{-}{I}_{{\rm{RCP}}}=2\cdot {\rm{Im}}({E}_{x}{{E}_{y}}^{\ast })$$

where *I*_*j*_ refers to the intensity of the polarization component projected in the *j* direction. For fully polarized light, *s*_0_^2^ = *s*_1_^2^ + *s*_2_^2^ + *s*_3_^2^, which allows all polarization states to be assigned a unique point on a sphere in (*s*_1_,*s*_2_,*s*_3_) space—the Poincaré sphere (Supplementary Fig. 10). Lines of constant *Ψ* are longitudes on the sphere. Just as globe longitudes intersect at the poles so that the longitudinal coordinate is undefined there, *Ψ* is singular at the Poincaré sphere poles at which circularly polarized light resides. There, *s*_1_ and *s*_2_ go to zero, in analogy to the vanishing of the real and imaginary parts of a scalar complex field at a phase singularity. It is noteworthy, however, that the overall field intensity at the polarization singularity can be non-zero, because *s*_3_ may not vanish.

**Fig. 5:**
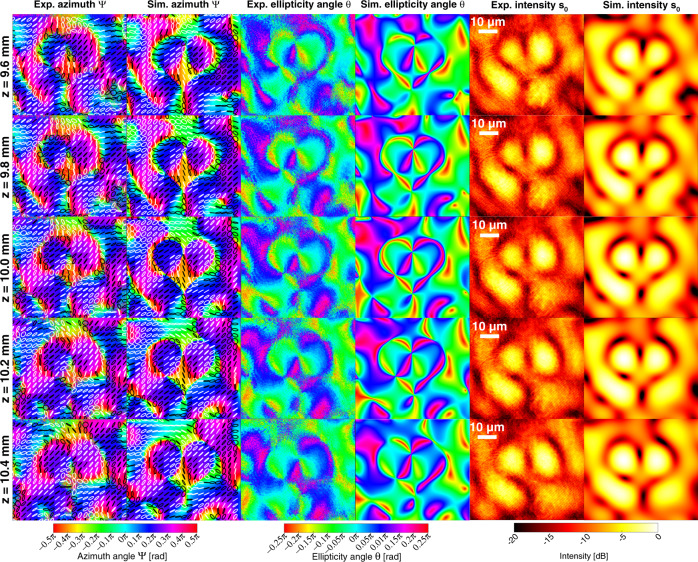
An engineered heart-shaped optical polarization singularity sheet (*λ*_*0*_ = 532 nm) profiled at various transverse planes. The columns are, from left to right, experimental polarization azimuth *Ψ*, simulated polarization azimuth *Ψ*, experimental ellipticity angle *θ*, simulated ellipticity angle *θ*, experimental intensity (*s*_0_ Stokes parameter) profile, and simulated intensity profile. Black and white ellipses in the azimuth plots indicate the local polarization ellipses. Black ellipses indicate left-handed elliptical polarization and white ellipses indicate right-handed elliptical polarization. The experimental optical field was produced by a polarization-sensitive metasurface. The images are captured at different displacements *z* from the metasurface along the propagating direction. The diagonal fringes in the experimental plots are artifacts arising from light diffracting around an iris in the optical path.

Figure 5 plots transverse cross-sections of an engineered polarization singularity sheet with a jump in *Ψ* tracing the shape of a heart (real and imaginary zero-isosurfaces of the *σ*-field are plotted in Supplementary Fig. 3). The cross-sectional heart-shape is identical to that of the phase-singularity sheet in Fig. 3, is also centered at *z* = 10 mm, and is designed to be generated by a plane wave at *λ*_*0*_ = 532 nm and with 45° linear polarization incident on a patterned 0.4 mm × 0.4 mm square aperture (at *z* = 0 mm) that serves as a spatially variant waveplate. The paraxial approximation (qualitatively supported by the target plane distance being much larger than the aperture size) is justified, as the energy density associated with the longitudinal *E*_*z*_ in the volume of interest is much smaller (in this case, <0.05%) than that of the transverse *E*_*x*_ and *E*_*y*_ components. The polarization singularity sheet is inversely designed by tuning the properties (phase delay, fast axis angle) of the spatially variant waveplate on a pixel-by-pixel level. The optimized parameters are plotted in Supplementary Fig. 11. Figure 5 plots *Ψ*, *θ*, and the field intensity (*s*_0_) of the simulated singularity sheet at and around *z* = 10 mm in the “Sim.” columns. The *Ψ* jump across the singularity has a magnitude of *π*/2 (as opposed to *π*, as in the phase-singularity sheet), because a sign flip in *σ* means that the change in arg(*σ*) = 2*Ψ* is *π*. The polarization singularity can also be visualized by examining the transverse gradient of the azimuth |∇_⊥_*Ψ*| (Supplementary Fig. 4), which displays rapid spatial variations far exceeding *k*_0_.

### Experimental realization of polarization singularity sheet

**Fig. 6:**
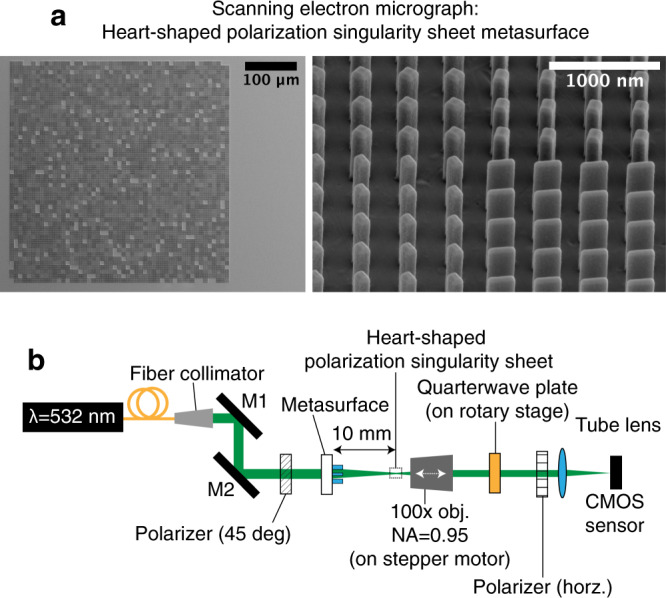
Fabrication and characterization of the heart-shaped polarization singularity metasurface. **a** Left, SEM image of the fabricated metasurface, which comprises 51 × 51 superpixels with a pitch of 8.4 μm. Each superpixel comprises 20 × 20 nanofins with a pitch of 0.42 μm. **a** Right, high-magnification SEM image at the interface of four pixels showing the individual nanofins. **b** Experimental setup for optical characterization of the 2D polarization singularity metasurface. The incident laser light is polarized at 45°. The transmitted light is magnified through a ×100 objective (numerical aperture (NA) = 0.95) before passing through a quarterwave plate and a horizontal analyzer. An image of the optical field is captured by a complementary metal oxide semiconductor (CMOS) camera sensor for every 5° of rotation of the quarterwave plate up to a total rotation angle of 180° from the horizontal. These images are used to reconstruct the polarization state of the optical field at a pixel-by-pixel level.

As designed, the polarization singularity requires a device that can control the polarization variation of an optical wavefront, point-by-point. Although a spatial light modulator can perform this by structuring and then superimposing two coherent beams with orthogonal polarizations, a single spatially variant waveplate is best suited for this application. One realization of a spatially variant waveplate is a polarization-sensitive metasurface. Transparent birefringent nanofins on a metasurface behave locally as waveplates that perform a unitary transformation of the incident electric fields^35^. We fabricated this metasurface using TiO_2_ nanofins on SiO_2_ (micrographs in Fig. 6a). The polarization singularity structures generated by the metasurface are analyzed at 41 *z-*positions around *z* = 10 mm using rotating quarterwave plate polarimetry^36^ in the experimental setup shown in Fig. 6b. The measurements of *Ψ*, *θ*, and intensity (*s*_0_) at and around *z* = 10 mm are plotted in Fig. 5 in the “Exp.” columns and show good agreement to the simulated patterns. The Stokes’ parameters comparison between simulations and experiment are plotted in Supplementary Fig. 12.

## Discussion

Here we provide experimental demonstrations of on-demand singularity shape engineering beyond simple curved or straight lines. We have achieved 2D singularity sheets with precisely engineered dark intensity profiles in both scalar and vector fields by maximizing phase gradients orthogonal to the desired sheet structures. On-demand singularity engineering opens up a vast set of possibilities in wide-ranging fields: the sensitivity of singularity sheets to perturbations and inhomogeneities in the propagation medium can be exploited to reconstruct density fluctuations and currents in transparent or weakly scattering media. This can be performed by sending a precisely engineered singularity structure into a weakly scattering medium, and then capturing the distorted light field with a phase-sensitive technique. The singularities may also be strategically introduced in holography to improve the light/dark contrast, or in microscopy and telescopy to selectively suppress signals from parts of the image, as an extension of the vortex coronagraph from astronomy^37,38^. Such shaped singularities may also be useful in conjunction with MINFLUX-like techniques to localize elongated fluorescent emitters (e.g., quantum rods), both in space and in rotation angles with deeply subwavelength precision. By rotating and translating a pump beam with an extended singularity feature around an elongated emitter, then finding the position and orientation that minimizes the fluorescent signal, one can identify the associated position and orientation of that elongated emitter with wavelength-independent precision. Singularity engineering may also be deployed in shaping radiofrequency or acoustic emission patterns so as to deterministically produce and manipulate dead or quiet zones.

In particular, singularity sheets may find application in atomic trapping of neutral atoms using intensity gradients. The vast majority of optical dipole traps for cold atoms are “red” traps, which trap atoms in arrays of tightly focused spots of light, where the light is red detuned from the dipole resonance. Blue traps with 3D spatial confinement, which trap the atoms in a dark spot surrounded by blue-detuned light, are much harder to realize and techniques using a single structured beam have only been able to produce single blue traps thus far^39–41^. Deterministic singularity engineering of singularities produced by a single incident beam and metasurface may prove to be useful in generating blue trap arrays or exotic shapes on demand.

Beyond optics, singularities are found in many complex wave systems, such as acoustic, particle beam, fluidic, and plasmonic systems. Similar strategies of optimizing spatial gradients may enable one to engineer complex fields imbued with the strange behavior of nearby singularities.

## Methods

Detailed methods for device optimization and fabrication are found in the Supplementary Methods.

### Simulations

Full vectorial field profiles were obtained by discretization of the vector diffraction propagation integrals^27^ on the Tensorflow automatic differentiation platform^42^. The free parameters for each optimization are either the phases at each pixel on a phase mask or the waveplate properties (phase shift in the fast and slow axes, rotation angle of the fast axis) at each pixel on a spatially varying waveplate. The optimization objective function to be maximized is a smooth approximation to a minimum function applied to the list of directional phase derivatives at specified points in the field. Optimization is performed using gradient descent through the Broyden–Fletcher–Goldfarb–Shanno algorithm^43^ with iterative refinement applied to the parameter distribution, to reduce the risk of converging to a poorly performing local optimum.

### Metasurface fabrication

The metasurfaces are fabricated out of TiO_2_ on a glass substrate^44,45^. The glass substrate is a 0.5 mm-thick JGS1-fused silica. The metasurface pattern is written into ZEP520A-positive electron-beam resist (thickness 600 nm) using a 125 kV electron-beam lithography system. After resist development and oxygen plasma descum, the patterned holes in the electron-beam resist are backfilled with amorphous TiO_2_ through atomic layer deposition up to a height of 200 nm above the resist patterns. The excess TiO_2_ is etched back using reactive ion etching until the resist surface is exposed and the residual resist is removed by immersion in Remover PG solution for 30 h, which leaves free-standing TiO_2_ nanopillars or nanofins.

### Device characterization

For the 2D phase-singularity sheet, 532 nm laser light is incident normally on the non-patterned face of the metasurface and the transmitted light is imaged by a microscope with a ×100 objective. The intensity image is captured at 41 *z*-planes, where *z* = 0 mm corresponds to the patterned surface of the metasurface. At each *z*-plane, the intensity image is captured at six different exposure times. These multiple exposure images are weighted by their respective exposure times and stacked to remove saturated pixels and produce a composite image with a large intensity dynamic range. The field phase at each *z*-plane was obtained by a modified version of the ﻿single-beam multiple-intensity reconstruction technique^31^.

For the 2D polarization singularity sheet, 532 nm light at 45° linear polarization is incident normally on the non-patterned face of the metasurface and the transmitted light is imaged by a microscope with a ×100 objective. Between the objective and the tube lens, we place a quarterwave plate and linear polarizer to act as a polarization analyzer. At each of the 41 *z*-planes, we capture 36 intensity images (3 exposure times for exposure-weighted image stacking) where the quarterwave plate fast axis is rotated in steps of 5°. We measure the four unnormalized Stokes parameters for each pixel using the rotating quarterwave plate method^36^.

## Supplementary information

Supplementary Information

## Data Availability

The data that support the findings of this study are available from the corresponding author upon reasonable request.

## Code availability

The code that support the findings of this study are available from the corresponding author upon reasonable request.
