# Supplementary material for: Engineering phase and polarization singularity sheets
Source: Nat Commun. 2021 Jul 7;12:4190. doi: 10.1038/s41467-021-24493-y (PMC8263812; doi:10.1038/s41467-021-24493-y)
Supplement: Supplementary file 1 — Supplementary Information [file 41467_2021_24493_MOESM1_ESM.pdf]

SUPPLEMENTARY INFORMATION

Engineering Phase and Polarization Singularity Sheets

AUTHORS

Soon Wei Daniel Lim[1*], Joon-Suh Park[1,2], Maryna L. Meretska[1], Ahmed H. Dorrah[1], Federico Capasso[1]*

[1]Harvard John A. Paulson School of Engineering and Applied Sciences, Harvard University, Cambridge, MA 02138, USA.

[2]Nanophotonics Research Centre, Korea Institute of Science and Technology, Seoul, 02792, Republic of Korea.

*Corresponding authors: lim982@g.harvard.edu and capasso@seas.harvard.edu

SUPPLEMENTARY METHODS

Production of 0D point singularities

One way to produce 0D point singularities is to place the singularities on the axis of a cylindrically symmetric field. Specifically, cylindrical symmetry here means that the complex electromagnetic field components (E_x, E_y, E_z, H_x, H_y, H_z) are only dependent on the radial distance r from the optical axis and the longitudinal coordinate z. Such a cylindrically symmetric field can be generated by uniform illumination of a patterned aperture that imposes a cylindrically symmetric phase profile. By minimizing the field amplitude or maximizing the z-directed phase gradient at the desired axial positions, one can produce 0D singularities at these positions.

In the demonstration system, we parametrize the cylindrically symmetric patterned aperture (located at z=-1000 μm) with 251 radial phase pixels, each spaced 4 μm apart in the radial direction, for a total aperture radius of 1000 μm. The incident vacuum wavelength is 532 nm and is linearly polarized in the x direction. We propagate the complex wavefront into the domain z>-1 mm using the vectorial diffraction integral. The objective function used for optimization is:

$$F = -\min\left\{\log\left[\left(\frac{\partial\phi}{\partial z_i}\right)^2\right]\right\}_{i=1,2,3} \tag{1}$$

Where $\frac{\partial\phi}{\partial z_i}$ is the axial phase gradient located at $z=z_i$. Minimizing F maximizes the z-directed phase gradient of the optical field at the three positions z_1=0 μm, z_2=1 μm, z_3=2 μm. To improve convergence, we use a smooth approximation to the minimum function, which has the benefit of being analytic instead of piecewise continuous:

$$\min(a_1, \dots, a_n) = \frac{\log[\sum_i \exp(-s \cdot a_i)]}{-s}, s = \frac{100}{\frac{1}{n}\sum_i |a_i|} > 0 \tag{2}$$

In this smooth approximation, the sum inside the logarithm will be dominated by the term corresponding to smallest value of a_i. s is a scale factor chosen to normalize the input array values and avoid numerical underflow or overflow during the computation of the exponential.

We perform the optimization using gradient descent, where the step size and termination condition are chosen through the Broyden–Fletcher–Goldfarb–Shanno (BFGS) algorithm[1].

Supplementary Fig. 2 plots the resultant field profile from the optimization. Supplementary Fig. 2a-b plot the field intensity (logarithmically-scaled) and phase as a function of (*r*,*z*). The *z*-directed propagation phase has been removed from the phase plot (by multiplication with exp(-*ikz*)) to show the long-scale phase variations. The three 0D singularities are visible as the low-intensity features along *r*=0 and *z*=0, 1, 2 µm, at the intersection of the real and imaginary zero-isolines. The radial phase mask on the patterned aperture that produces this singularity pattern is plotted in Supplementary Fig. 2c.

Optimization details for the 2D heart-shaped phase singularity

The phase-controlled surface at *z*=0 is parametrized with a grid of 101×101 superpixels, each spaced 8 µm apart in the x and y directions. For each superpixel, we assign a value of ϕ for the propagation delay for light incident at that position. We then illuminate the phase-controlled surface with a uniform plane wave of unit intensity and vacuum wavelength 532 nm. This produces a complex wavefront at *z*=0, which we propagate into the domain *z*>0 using the vectorial diffraction integral.

At the focal distance of $z = f = 10$ mm, the heart-shaped singularity boundary is described by a parametric curve[2]:

$$x(t) = 16\, s \sin^3 t\, , y(t) = s(13 \cos t - 5 \cos 2t - 2 \cos 3t - \cos 4t), t \in (0,2\pi) \quad (3)$$

The curve positions are scaled by the scale parameter $s = 1$ µm and centered at the origin so that the heart centroid lies at the origin in the xy plane. A total of 50 values of t linearly spaced between 0 and 2π (excluding 0 and 2π) are used to parametrize the curve $\{(x_i, y_i)\}, i = 1, \ldots, 50$. For each point (x_i, y_i) on the curve, the inward-directed normal vector $\hat{n}_i$ is also computed:

$$\hat{n}_i = \frac{\left(-y'(t_i), x'(t_i)\right)}{\sqrt{x'(t_i)^2 + y'(t_i)^2}} \quad (4)$$

The normal vector is used to compute the directional derivative of the field propagation phase gradient $\frac{\partial \phi}{\partial n_i}$:

$$\frac{\partial \phi}{\partial n_i} = \nabla_\perp \phi \cdot \hat{n}_i = \left(\frac{\partial \phi}{\partial x}, \frac{\partial \phi}{\partial y}\right) \cdot \hat{n}_i \quad (5)$$

The objective function is the minimum of the squares of the directional derivatives at each point on the heart:

$$F = -\min\left\{\left(\frac{\partial \phi}{\partial n_i}\right)^2\right\}_{i=1,\dots,50} \tag{6}$$

We minimize F during the optimization so that the directional phase derivatives over the singularity boundary are maximized. To improve convergence, we use a smooth approximation to the minimum function.

We perform the optimization using gradient descent with the BFGS algorithm. To reduce the risk of getting stuck in a local minimum of the objective function, we implement an iterative refinement scheme. The optimization is performed at three spatial resolutions, beginning with the lowest spatial resolution mesh (largest spatial grid size). Upon convergence, the optimized solution $\phi_x(x_s, y_s)$ at the lower spatial resolution is linearly interpolated onto a higher spatial resolution mesh as the starting condition, and the optimization is run again using the higher spatial resolution mesh. The spatial resolution in x and y is improved by a factor of 1.3 at each iteration and a total of three iterations is performed, including the final iteration with the desired highest spatial resolution. The starting point for the lowest spatial resolution mesh is selected by randomly sampling 100 configurations where each phase pixel ϕ_x is drawn from a uniform distribution between $[-\pi, \pi]$, and then selecting the configuration with the best objective function value.

In order to realize the optimized phase profile in a metasurface that operates in transmission, we discretize each of the 101x101 superpixels (pitch 8 μm) into a 32x32 grid, where the smaller grid has a subwavelength pitch of 0.25 μm. Each grid square is associated with the phase of the larger 8 μm superpixel from which it was formed. Under the locally periodic assumption and the unit cell approach, we seek to place a nanostructure (meta-atom) at each grid square to enforce the required phase at that position. This nanostructure is chosen from a "library" of meta-atoms with pre-computed optical properties, where the numerical simulation was performed under the assumption that the meta-atoms are periodic in the transverse plane. Since the heart-shaped phase singularity can be realized in a scalar field, the polarization of light is not important, and we chose to work with polarization-insensitive cylindrical meta-atoms to build the meta-atom library. The nanopillars are made of 600 nm-tall amorphous Titanium dioxide (TiO_2, n=2.40 at 532 nm), spaced 0.25 μm apart in a square lattice, and mounted on a substrate of fused silica (n=1.46 at 532 nm). These meta-atoms are exhibited schematically in Supplementary Fig. 13a.

For each nanopillar diameter, we simulate a uniform array of identical nanopillars using the RETICOLO package for Rigorous Coupled Wave Analysis[3] to obtain the zeroth-order transmitted phase. The circular cross-sections of the nanopillars are discretized using a staircase approximation with 800 points over the circumference, and 81 x 81 Fourier harmonics are used in the x and y directions on the plane. The dependence of the phase and transmission efficiency as a function of the nanopillar diameter is plotted in Supplementary Fig. 13b. Nanopillars of diameter between 60 nm and 215 nm provide 2π phase coverage and are thus used to construct the metasurface based on the required phase profile from optimization.

Optimization details for the 2D heart-shaped polarization singularity

The metasurface at $z = 0$ is parametrized with a grid of 51 x 51 superpixels, each spaced 8.4 nm apart in the x and y directions. We treat the metasurface as a spatially varying wave plate which manipulates the local polarization state[4,5]. This allows us to assign three values for each meta-element position on the metasurface: ϕ_x (for the propagation delay for light polarized along the fast axis of the meta-element), ϕ_y (for the propagation delay for light polarized orthogonal to the fast axis) and θ_{rot} (for the overall rotation angle of the meta-element fast axis). The spatially varying Jones matrix of the meta-surface can then be written as:

$$J(x_s, y_s) = R\big(-\theta_{rot}(x_s, y_s)\big) \begin{pmatrix} \exp i\phi_x(x_s, y_s) & 0 \\ 0 & \exp i\phi_y(x_s, y_s) \end{pmatrix} R\big(\theta_{rot}(x_s, y_s)\big) \tag{7}$$

where $R(\theta_{tot})$ is the 2×2 rotation matrix.

We then illuminate the phase-controlled surface with a uniform plane wave of unit intensity and wavelength 532 nm polarized in the $(\hat{x} + \hat{y})$ diagonal direction. This produces a wavefront of transverse electric field values at $z = 0$, which we propagate into the domain $z > 0$ using the vectorial diffraction integral. We consider all three Cartesian components of the electric field after propagation.

One can calculate the polarization azimuth by determining the direction of maximal oscillation exhibited by the transverse electric field. At a position in the transverse plane, the direction of the transverse electric field can be written as:

$$\boldsymbol{E} = E_x e^{-i\omega t}\, \hat{x} + E_y e^{-i\omega t} \hat{y} \tag{8}$$

E_x and E_y are complex phasors encoding the amplitude and phase of the Cartesian electric fields. The time-average of each individual Cartesian component is zero over one optical cycle.

The polarization azimuth is calculated from the Stokes parameters of the electric field:

$$s_0 = |E_x|^2 + |E_y|^2 \tag{9}$$

$$s_1 = |E_x|^2 - |E_y|^2 \tag{10}$$

$$s_2 = 2\,\mathrm{Re}(E_x E_y^*) \tag{11}$$

$$s_3 = 2\,\mathrm{Im}(E_x E_y^*) \tag{12}$$

The normalized Stokes parameters are obtained by dividing through by s_0, the intensity component.

$$S_1 = \frac{s_1}{s_0}, S_2 = \frac{s_2}{s_0}, S_3 = \frac{s_3}{s_0} \tag{13}$$

The complex σ field is constructed using the normalized Stokes parameters as $\sigma = S_1 + iS_2$, noting that all the Stokes parameters are real numbers. The polarization azimuth is then half the complex angle of the σ field.

$$\Psi = \frac{1}{2}\arg\sigma \tag{14}$$

One should recognize the σ field as proportional to the time average of $E_x + iE_y$:

$$\sqrt{\left\langle \left(E_x + iE_y\right)^2 \right\rangle} = \sqrt{\langle E_x^2 \rangle - \langle E_y^2 \rangle + 2i\langle E_x E_y \rangle}$$

$$= \sqrt{\frac{1}{2}|E_x|^2 - \frac{1}{2}|E_y|^2 + i\Re(E_x E_y^*)}$$

$$= \sqrt{\frac{1}{2}(s_1 + is_2)}$$

$$= \sqrt{\frac{s_0}{2}}\sqrt{\Sigma_{12}}$$

$$\Rightarrow \arg\sqrt{\left\langle \left(E_x + iE_y\right)^2 \right\rangle} = \arg\sqrt{\sigma} = \frac{1}{2}\arg\sigma = \Psi \tag{15}$$

For linear polarization, the polarization azimuth is the direction of polarization relative to the transverse x-axis.

At the focal distance of f = 10 mm, the heart-shaped singularity boundary is described by a parametric curve[2]:

$$x(t) = 16\, s \sin^3 t\,, y(t) = s(13 \cos t - 5 \cos 2t - 2 \cos 3t - \cos 4t), t \in (0,2\pi) \qquad (16)$$

The curve positions are scaled by the scale parameter s = 1 μm and centred at the origin so that the heart centroid lies at the origin in the xy plane. A total of 50 values of t linearly spaced between 0 and 2π (excluding 0 and 2π) are used to parametrize the curve $(x_i, y_i), i = 1, \dots, 50$. For each point (x_i, y_i) on the curve, the inward-directed normal vector $\hat{n}_i$ is also computed:

$$\hat{n}_i = \frac{\left(-y'(t_i), x'(t_i)\right)}{\sqrt{x'(t_i)^2 + y'(t_i)^2}} \qquad (17)$$

The normal vector is used to compute the directional derivative of the field polarization azimuth $\frac{\partial \Psi}{\partial n_i}$:

$$\frac{\partial \Psi}{\partial n_i} = \nabla_\perp \Psi \cdot \hat{n}_i = \left(\frac{\partial \Psi}{\partial x}, \frac{\partial \Psi}{\partial y}\right) \cdot \hat{n}_i \qquad (18)$$

The objective function is the minimum of the squares of the directional derivatives at each point on the heart:

$$F = -\min \left\{ \left(\frac{\partial \Psi}{\partial n_i}\right)^2 \right\}_{i=1,\dots,50} \qquad (19)$$

We minimize F during the optimization so that the directional phase derivatives over the singularity boundary are maximized. To improve convergence, we use a smooth approximation to the minimum function.

To produce a metasurface that is able to achieve the required spatially variant waveplate behaviour, we use the locally periodic assumption with the unit cell approach. Each of the 51 x 51 superpixels (pitch 8.4 μm) is partitioned into a 20 x 20 grid with subwavelength pitch of 420 nm. We associate the required (φ_x, φ_y, θ_{rot}) waveplate parameters of the larger pixel with the smaller grid positions. For each grid position, we pick the meta-atom from a pre-computed library that most closely satisfies the required (φ_x, φ_y, θ_{rot}) waveplate parameters. The meta-atom library was provided by Dr Noah Rubin and was described in a previous publication[6]. The meta-

atoms are rectangular nanofins made of 600 nm tall amorphous Titanium dioxide with a pitch of 420 nm. These meta-atoms are exhibited schematically in Supplementary Fig. 13c and are parametrized by the width of the nanofin in the x and y transverse directions. The dependence of the phase delay along the x-direction of the meta-atom and the transmission efficiency on each of these x and y transverse widths are plotted in Supplementary Fig. 13d and e, respectively.

Characterization of the 2D heart-shaped phase singularity metasurface

Fig. 4a exhibits scanning electron micrographs of a metasurface processed under identical conditions to the metasurface used for optical characterization. The metasurface used for optical characterization was not imaged in the SEM because this requires the irreversible deposition of a conductive metallic layer. The experimental setup for characterizing the 2D heart-shaped phase singularity metasurface is exhibited in Fig. 4b. 532 nm laser light (Ventus 532, 30 GHz spectral bandwidth) from a single-mode fibre is collimated by a reflective collimator (Thorlabs RC04APC-P01, 4 mm collimated beam diameter), aligned by two mirrors (M1 and M2 in Fig. 4b) before being incident normally on the non-patterned glass face of the metasurface. The transmitted light is imaged with a 100x infinity-corrected objective (Olympus MPLAPON 100x, NA 0.95) and tube lens (Thorlabs TTL-180A) before being captured by a monochromatic CMOS camera (Thorlabs DCC1545M, 1280 x 1024 pixels, 5.2 μm pixel pitch). The metasurface z-position is controlled by a motorized translation stage (Thorlabs DRV208). The intensity image is captured at 41 z-positions from z=9.6 mm to z=10.4 mm with steps of 0.02 mm, where z=0 mm corresponds to the patterned surface of the metasurface. At each z-position, the intensity image is captured at 6 different exposure times ranging from 5 ms to 984 ms. These multiple exposure images are later weighted by their respective exposure times and stacked to remove saturated pixels and produce a composite image with a large intensity dynamic range. This step is essential in visualizing the low intensity profile of singular regions and compensates for the limited dynamic range of a single 8-bit intensity capture, which can only yield brightness values at integer values between 0 and 255 inclusive.

The phase of the optical field at each of the 41 z-positions was obtained by a modified version of the single-beam multiple-intensity reconstruction (SBMIR) technique[7]. This process is depicted schematically in Fig. 4c. This method involves repeated application of Gerchberg-Saxton forward and backward propagation between pairs of intensity captures at different z-positions. We downsample the images by a factor of 10 in both horizontal and vertical directions to speed up the calculation process. We use a cycle starting with the image at z=9.6 mm (image 1),

forward propagating to z=9.62 mm (image 2), backward propagating back to image 1, forward propagating to image 3, back to image 1 and repeating this for each consecutive image until image 41. During each propagation step, we use the true intensity profile (captured experimentally) and the retrieved phase profile as the initial boundary conditions for the propagation. The resultant forward or backward propagated field phase at the target plane then becomes the updated retrieved phase at the target plane. The starting retrieved phase estimated for all planes is set to zero. We repeat this cycle 50 times. The forward propagation is performed using the x-polarization component of a full vectorial propagator. The backward propagation is performed by replacing the wavevector k in the forward propagator with $-k$. The accuracy of the phase retrieval is measured by the root-mean-squared (RMS) deviation between the estimated intensity (each normalized by their respective maximum intensities) after a propagation step and the true intensity map at that plane. We observe that by the second cycle, the maximum RMS deviation over all forward and backward propagation steps within the cycle remains between 6.2% to 6.5% for every cycle from cycle 4 onwards.

Characterization of the 2D heart-shaped polarization singularity metasurface

Fig. 6a exhibits scanning electron micrographs of a metasurface processed under identical conditions to the metasurface used for optical characterization. The metasurface used for optical characterization was not imaged in the SEM because this requires the irreversible deposition of a conductive metallic layer. Fig. 6b exhibits the experimental setup used for optical characterization of the metasurface that produces a 2D heart-shaped polarization singularity. 532 nm laser light (Ventus 532, 30 GHz spectral bandwidth) from a single-mode fibre is collimated by a reflective collimator (Thorlabs RC04APC-P01, 4 mm collimated beam diameter), aligned by two mirrors (M1 and M2), polarized to 45°, before being incident normally on the unpatterned glass face of the metasurface. The transmitted light is imaged with a 100x infinity-corrected objective (Olympus MPLAPON 100x, NA 0.95) and tube lens (Thorlabs TTL-180A) before being captured by a monochromatic CMOS camera (Thorlabs DCC1545M, 1280 x 1024 pixels, 5.2 µm pixel pitch). Between the objective and the tube lens, we place a zero-order quarterwave plate (Thorlabs WPQ10M-532) and a wire-grid polarizer (axis horizontal to the optical table, taken to be the x direction) to act as an analyser. The quarterwave plate is mounted on a motorized rotary stage (Thorlabs K10CR1). The objective z-position is controlled by a motorized translation stage (Thorlabs Z825B). At each of the 41 z-positions from z=9.6 mm to z=10.4 mm (steps of 0.02 mm), where z=0 mm corresponds to the patterned surface of the metasurface, we capture 36 intensity images where the quarterwave plate fast axis is rotated from

0 degrees (aligned to the x-direction) to 175 degrees, in steps of 5 degrees. At each quarterwave plate angle, we capture the intensity image at 3 different exposure times ranging from 100 ms to 980 ms. These multiple exposure images are later weighted by their respective exposure times and stacked to remove saturated pixels and produce a composite image with a large intensity dynamic range.

For each of the 41 z-positions, we measure the four unnormalized Stokes parameters (s_0, s_1, s_2, s_3) based on the 36 images captured at each of the quarterwave plate rotation angles. We use the rotating quarterwave plate method introduced by Schaefer et. al.[8] to write the transmitted light intensity as a function of the quarterwave plate rotation angle θ_{QWP} as:

$$I(\theta_{QWP}) = \frac{1}{2}\left(A + B\sin 2\theta_{QWP} + C\cos 4\theta_{QWP} + D\sin 4\theta_{QWP}\right) \tag{20}$$

Where, $A = s_0 + s_1/2$, $B = s_3$, $C = s_1/2$, $D = s_2/2$. We thus extract the intensity variation of each pixel as the quarterwave plate is rotated and fit the intensity variation-angle relationship to that exhibited above, where A, B, C, and D are the fitting parameters. The polarization azimuth can then be computed using the four-quadrant arctangent $2\Psi = \text{atan2}(s_2, s_1)$ and the polarization ellipticity angle can be obtained using $2\theta = \sin^{-1}(-s_3/s_0)$. The fitted Stokes parameters at the plane z = 10 mm are displayed in Supplementary Fig. 12e-h and are compared to the numerically simulated Stokes parameters on the same plane in Supplementary Fig. 12a-d, exhibiting excellent agreement.

SUPPLEMENTARY NOTES

1. Mathematical relationship between phase gradients and singularities

Here, we elucidate the relationship between phase gradients and complex scalar field zeros (singularities). Consider a complex scalar field E(**r**), which can be written in polar coordinates as $E(\mathbf{r})=A(\mathbf{r})\exp[i\phi(\mathbf{r})]$, $A(\mathbf{r})\in\mathbb{R}$. The phase gradient is $\nabla\phi(\mathbf{r})=\text{Im}[\nabla E(\mathbf{r})/E(\mathbf{r})]$. If a field can be written as a finite sum of plane waves, then the magnitude of the field and its derivatives are bounded, and therefore having an infinite phase gradient $\nabla\phi(\mathbf{r_0})$ at a point $\mathbf{r_0}$ implies that there is vanishing $E(\mathbf{r_0})$ at the same location – a singularity. However, the reverse implication is not true – vanishing field intensities do not imply infinite phase gradients: if the field gradient $\nabla E(\mathbf{r})$ converges to zero as $\mathbf{r}\rightarrow\mathbf{r_0}$ as a linear function of E(**r**), the phase gradient $\nabla\phi(\mathbf{r})$ does not diverge when $\mathbf{r}\rightarrow\mathbf{r_0}$. An example of such a reverse implication can be found in systems with purely real $E(\mathbf{r})$, such as a standing wave pattern of two counterpropagating plane waves, where the phase is

a constant everywhere by definition, and thus zeros in the field all exhibit a zero phase gradient as well. Apart from this special case, however, in most complex wave systems, it is more common that there exists some direction of approach $\mathbf{r} \rightarrow \mathbf{r_0}$ such that $\nabla E(\mathbf{r})$ and $E(\mathbf{r})$ have a complex phase difference. $E(\mathbf{r})$ thus exhibits an infinite phase gradient in that direction. In these cases, an infinite phase gradient is synonymous with an optical phase singularity.

2. Why a 3D volumetric optical singularity is not possible

A 3D optical singularity such as a "ball" of darkness is not physically possible. To see why, let us assume that one such singularity exists in a non-trivial field distribution (i.e., the field is not zero everywhere). By the definition of a singularity, the field and all its directional derivatives must be identically zero inside its non-zero 3D volume. An analytic field, such as an electromagnetic field, can be expressed with a series function, such as a Taylor expansion, about a point in its occupying space. If that point is within the 3D singularity that the field is zero, the field expansion across the space must be equal to zero, which contradicts the initial assumption of 3D singularity in a non-trivial field distribution. Thus, a 3D singularity cannot exist mathematically. However, it is possible to create optical fields where the field value is very low and approaches zero in a finite volume, such as in the case of a "perfect" optical vortex with a hollow (dark) center[9]. Such cases are approximate but not true mathematical 3D singularities.

3. Singularity shaping with the Gerchberg-Saxton algorithm

We deploy a Gerchberg-Saxton algorithm (GS) to design dark patterns and compare the results against that obtained through phase gradient maximization. The GS algorithm follows these steps:

1. Initialize the starting pixelated plane (metasurface) with zero phase and unit field intensity.
2. Initialize the target plane with zero phase and a target pixelated intensity pattern.
3. Forward propagate the starting plane fields to the target plane (located a distance $z>0$) away using the x-directed component of the fully vectorial Green's function[27].
4. At the target plane, compute the pixel-by-pixel standard deviation between the previous target plane phase pattern and the forward propagated field phase.
5. Replace the target plane phase pattern with that from the forward propagated field.
6. Reverse propagate (replacing k with $-k$) the target plane fields from the target plane back to the starting plane using the x-directed component of the fully vectorial Green's function.

7. At the starting plane, compute the pixel-by-pixel standard deviation between the previous starting plane phase pattern and reverse propagated field phase.
8. Replace the starting plane phase pattern with that from the reverse propagated field.
9. Check if the standard deviation values from step (4) and (7) are both smaller than an acceptable tolerance. If so, terminate. If not, return to step (3) and iterate.
10. The final phase profile at the starting plane is the desired phase profile that generates the target intensity pattern at the target plane.

To give the GS algorithm the best conditions to produce dark patterns, we consider two approaches. The first approach uses a binary target pattern in which the target field intensity is either zero or unity. The results of this study are exhibited in Supplementary Fig. 8. For all these GS designs, we keep the starting plane parameters fixed (aperture size 0.8 mm x 0.8 mm, phase pixel pitch 8 μm, incident wavelength 532 nm) and also fix the propagation distance to the target plane at 10 mm. This matched the geometry used to design the 2D phase singularity sheets. As a baseline, we consider using the GS algorithm to design a bright heart outline against a dark background, which is close to the standard use cases of the algorithm. We observe that when the heart shape and outline line thickness is large, the GS algorithm faithfully replicates the desired intensity pattern. However, as the target heart intensity pattern is reduced in size towards the scale used in the phase gradient maximization method, the fidelity of this replication becomes poorer, and the heart-shaped outline cannot be made as thin as that of the target pattern. In fact, the thickness of the GS-optimized heart-shaped outline closely matches the diameter of the first Airy disk minimum ($1.22\lambda_0$/NA, or 11.5 μm for the geometry examined) on the target plane, for an Airy disk generated by a perfect lens of diameter equal to the square aperture diagonal, focusing light onto the target plane. The physical mechanism for this is that the target plane intensity profile is generated by a superposition of waves that have a *bandlimited* transverse spatial frequency. To a good approximation, any field pattern on the target plane is generated by a complex superposition of plane waves emanating from the aperture. These plane waves have different transverse spatial frequencies based on the angle of incidence onto the target plane, with a transverse wavenumber $k_\perp$ that is dependent on the angle of incidence from the normal θ, $k_\perp = k_0 \sin\theta$. The target plane field pattern is thus a bandlimited superposition of these plane waves with different $k_\perp$, with the bandlimits corresponding to the extremal plane waves with the largest angles of incidence and hence the shortest periodicities on the transverse target plane. The Airy disk diameter is proportional to the shortest periodicity (specifically, 1.22 times) and thus sets an estimate for the minimum feature size on the target plane. The smallest linewidths on the target plane as obtained through the GS algorithm appear to be limited by this minimum

periodicity. In order to achieve feature sizes that are smaller than the shortest periodicity in a bandlimited function, one has to construct superoscillatory functions[10], which require specialised modifications to the GS algorithm, such as additional custom filters[11], or gradient-free high dimensional optimization techniques[12].

We perform the same GS optimization for a dark heart-shaped outline against a bright background as well, and these results are displayed in the lower two rows of Supplementary Fig. 8. Unlike the case of the bright heart-shaped outline, the dark intensity patterns produced by the GS algorithm exhibit much poorer replication fidelity, breaking apart into discontinuous dark patches as the target heart-shaped pattern scales down in size. Again, the minimum feature size of bright spots in the achieved intensity pattern is similar in scale to the Airy disk diameter. Since the bright background occupies the majority of the target intensity pattern, and the GS algorithm weights each pixel equally, and the GS algorithm acts to minimize the intensity deviation over a transverse area, it prioritises replicating this uniformly bright background at the expense of the dark heart-shaped features.

A major issue facing the GS design of a phase mask to attain a binary target pattern is that the binary pattern cannot be replicated perfectly since the binary pattern is not a solution to the electromagnetic wave equation. We can improve the behaviour of a GS-optimized phase mask by specifying a target intensity pattern that is *a priori* known to be part of a valid wave equation solution. This is the second GS design approach that we undertook. Instead of using a binary intensity pattern, we use the actual heart-shaped phase singularity sheet intensity pattern of Fig. 3d on the target plane, which is known to be a valid wave solution since it was designed using the phase gradient maximization technique. Performing the same GS algorithm with this non-uniform intensity mask, we obtain the target plane intensity and phase patterns in Fig. 3f and 3i, respectively. Although these target plane patterns more closely replicate the desired heart-shaped dark outline as compared to the binary target patterns in Supplementary Fig. 8, they still do not achieve the fidelity of the phase gradient maximization technique. Fig. 3f and 3i thus exhibit the best performing dark patterns attained through two GS approaches, which still pale in comparison to that attainable through phase gradient maximization.

Calculating topological charge in the transverse plane

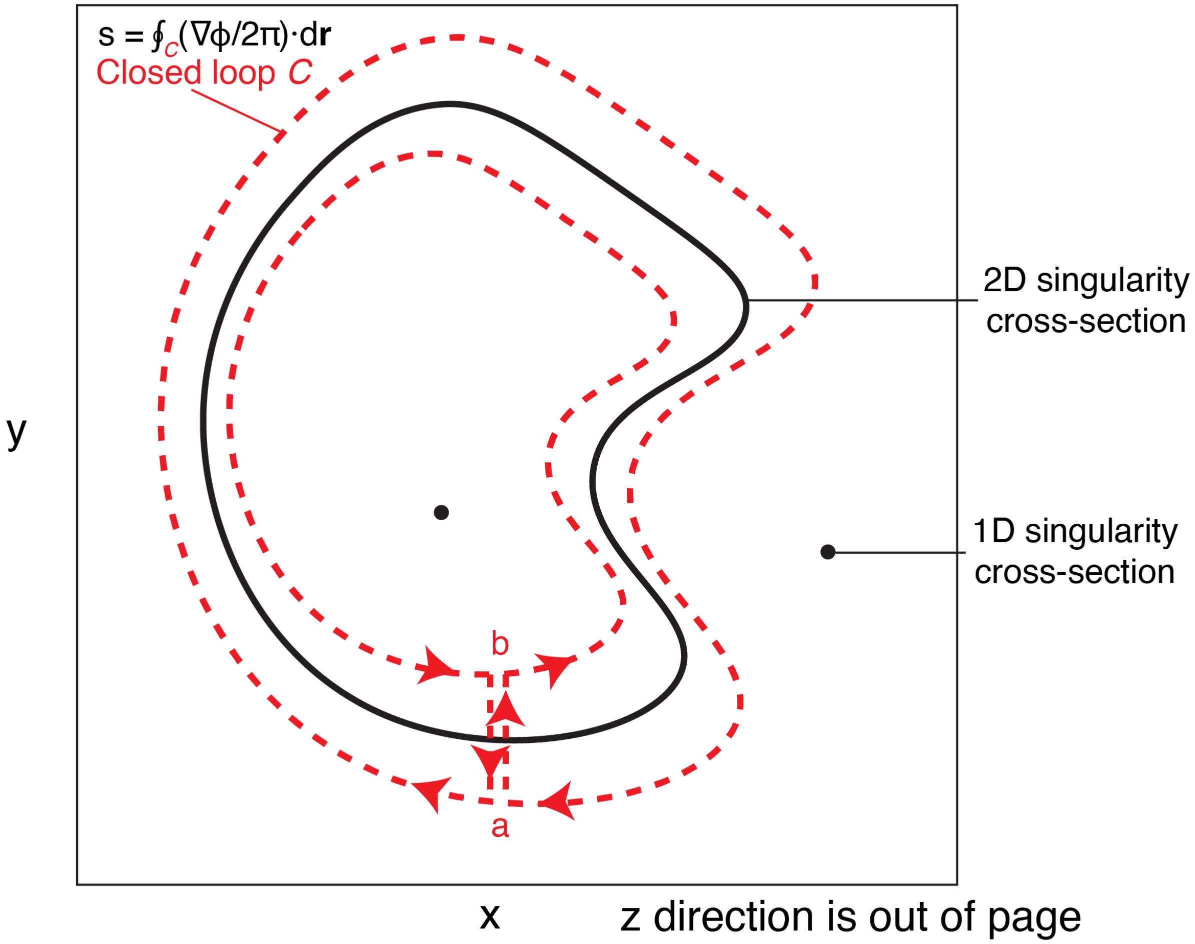

Supplementary Figure 1. **Closed loop to compute the topological charge of a 2D singularity with closed transverse cross-section**. Black dots and solid lines represent the loci of 1D and 2D singularity cross-sections on the transverse plane, respectively. In order to compute the topological charge of the 2D singularity with a closed cross-section without including the influence of the 1D singularity it encircles, we define a closed loop C that comprises an anticlockwise inner loop and a clockwise outer loop connected by a cut ab. Although the cut traverses the singularity cross-section, since it is traversed in both directions, its contribution to the line integral is zero. The topological charge $s=\oint_C(\nabla\phi/2\pi)\cdot d\mathbf{r}$ computed along C is the conserved charge associated with the 2D singularity.

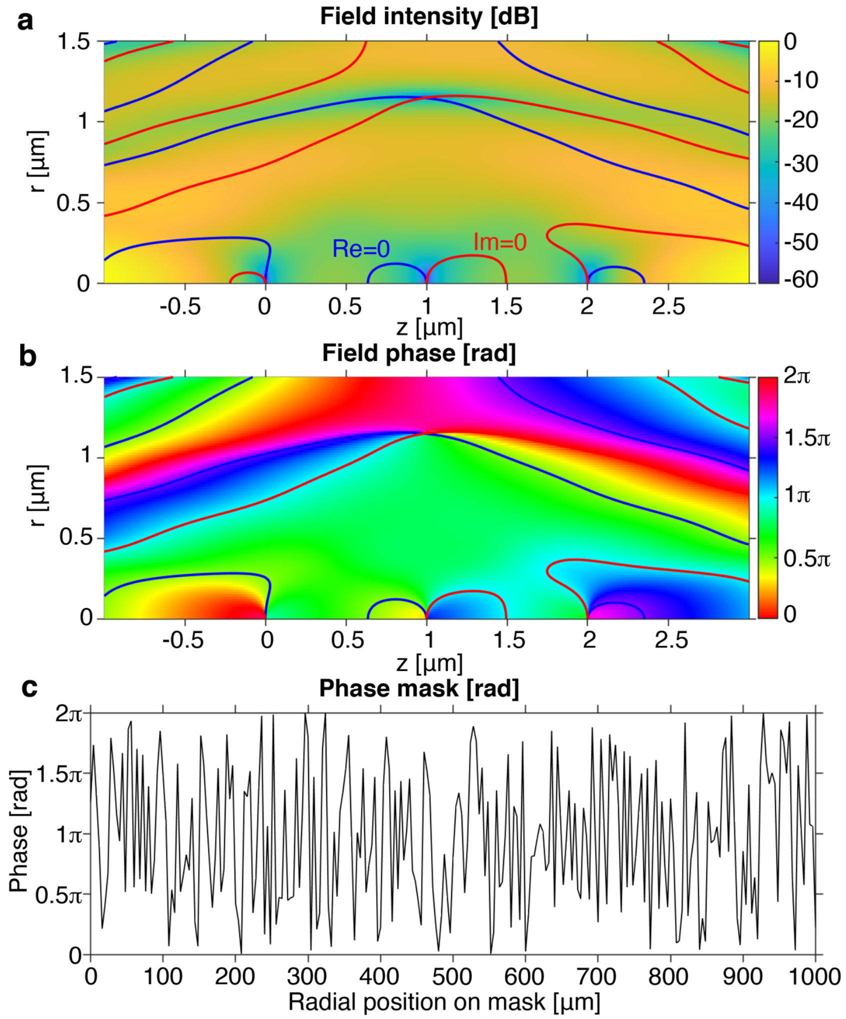

Supplementary Figure 2. **Three 0D point singularities in a cylindrically symmetric field with radial coordinate *r* and axial coordinate *z*.** **a**, Intensity and **b**, phase profiles of the field. The 0D singularities are located at z = 0, 1, and 2 μm along the optical axis (r = 0 μm). The blue contour is the isoline at which the real part of the field vanishes and the red contour is the isoline on which the imaginary part of the field vanishes. The complex scalar field has been multiplied by exp(-ikz) to remove the rapidly varying propagation phase. **c** Radial phase profile of the cylindrically symmetric phase mask placed at z = -1 mm that generates the three 0D singularities upon illumination with λ_0=532 nm light.

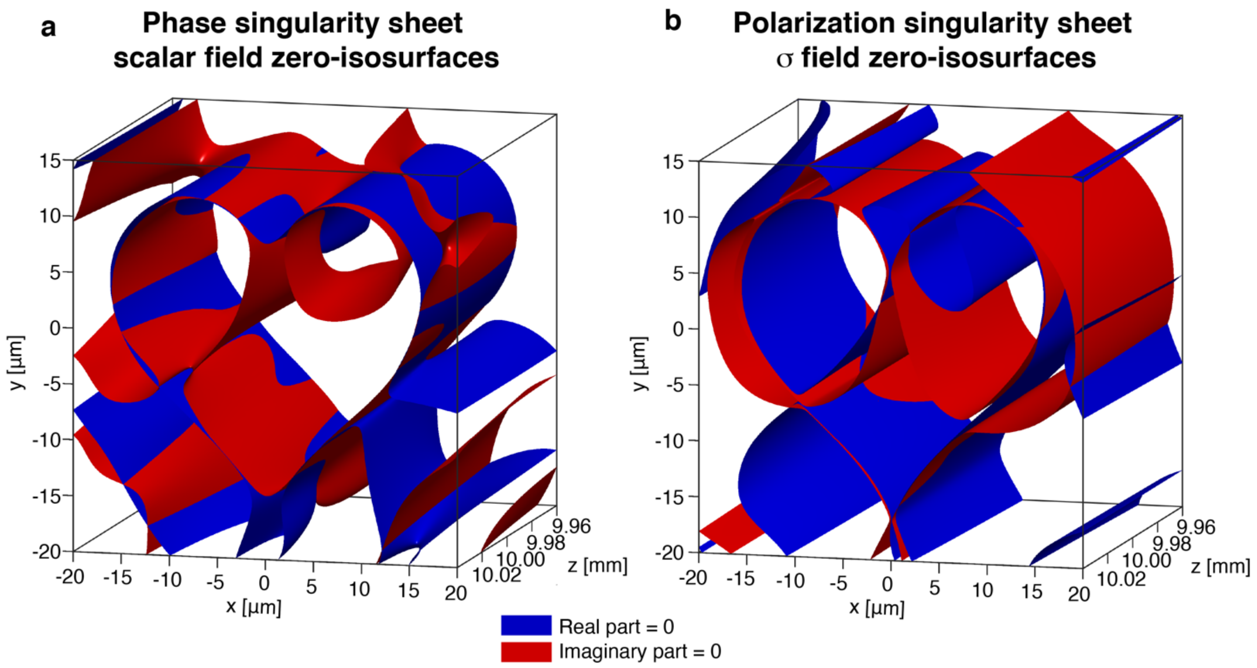

Supplementary Figure 3. **Real and imaginary zero-isosurfaces of the heart-shaped singularity sheets. a** Real and imaginary zero-isosurfaces of the scalar field of the heart-shaped phase singularity sheet and **b**, the field $\sigma = s_1 + is_2$ of the heart-shaped polarization singularity sheet.

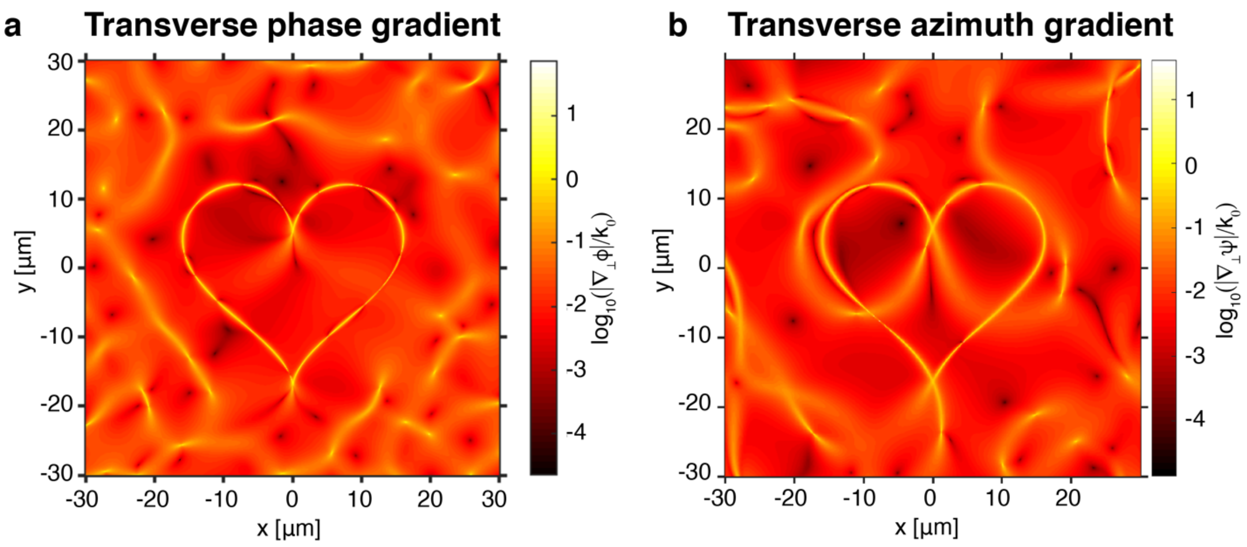

Supplementary Figure 4. **Numerically simulated transverse phase and azimuth gradients for heart-shaped singularity sheets, normalized to the incident wavenumber k_0.** **a** Plot of the magnitude of the transverse phase gradient at the z=10 mm plane for the 2D phase singularity sheet, $|\nabla_\perp \phi|=[(\partial_x \phi)^2+(\partial_y \phi)^2]^{1/2}$. **b** Plot of the magnitude of the transverse polarization azimuth gradient at the z=10 mm plane for the 2D polarization singularity sheet, $|\nabla_\perp \Psi|=[(\partial_x \Psi)^2+(\partial_y \Psi)^2]^{1/2}$. Note that the transverse gradient can far exceed the wavenumber in both cases, indicating superoscillatory behaviour.

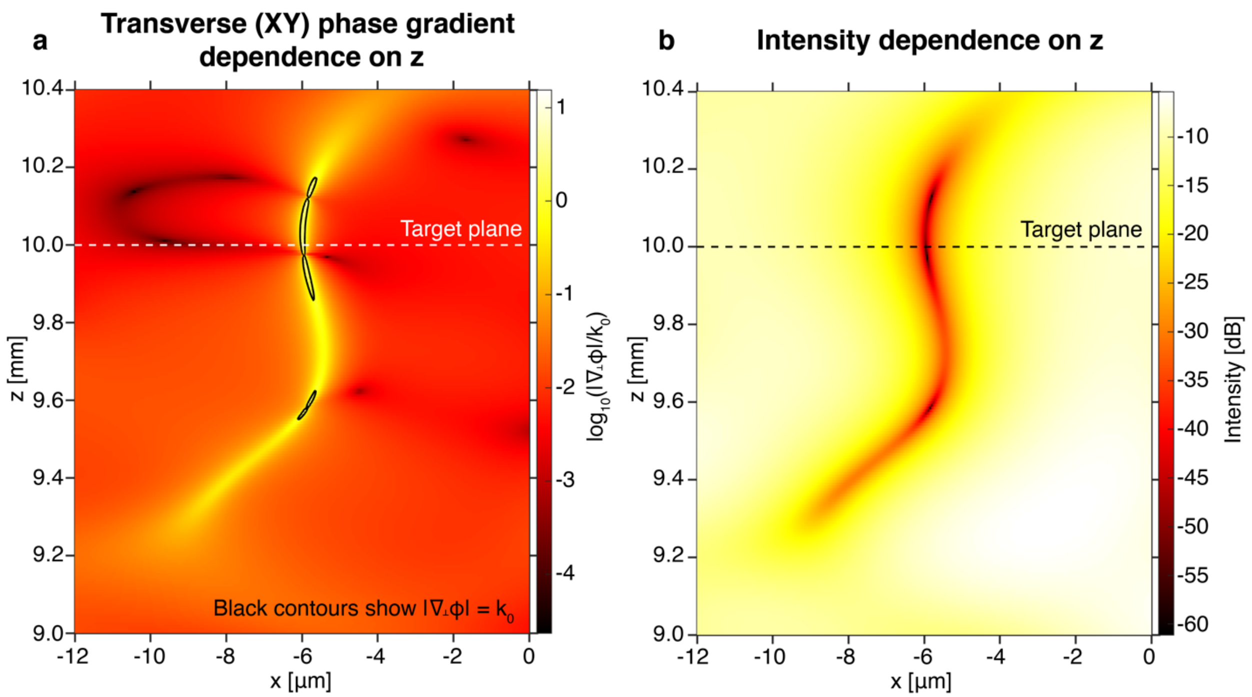

Supplementary Figure 5. **Longitudinal (*z*) variation of transverse phase gradients and intensity for the heart-shaped optical phase singularity sheet.** These plots correspond to the longitudinal variation along the 1D cut profile line in Fig. 3d. The dotted lines at z=10 mm indicate the position of the target plane at which the phase gradients were optimized. **a** Magnitude of the transverse phase gradient $|\nabla_{\perp}\phi|=[(\partial_x\phi)^2+(\partial_y\phi)^2]^{1/2}$ as a function of transverse coordinate x and longitudinal coordinate z. The black contours encircle the superoscillatory region where $|\nabla_{\perp}\phi|\geq k_0$, the incident light wavenumber. The superoscillatory region extends approximately 300 wavelengths in front and behind of the target plane. **b** Intensity as a function of x and z. The intensity scale is identical to that of Fig. 3d, which is normalized to the maximum intensity over the z=10 mm plane. The low intensity region is coincident with the region of large phase gradient magnitudes.

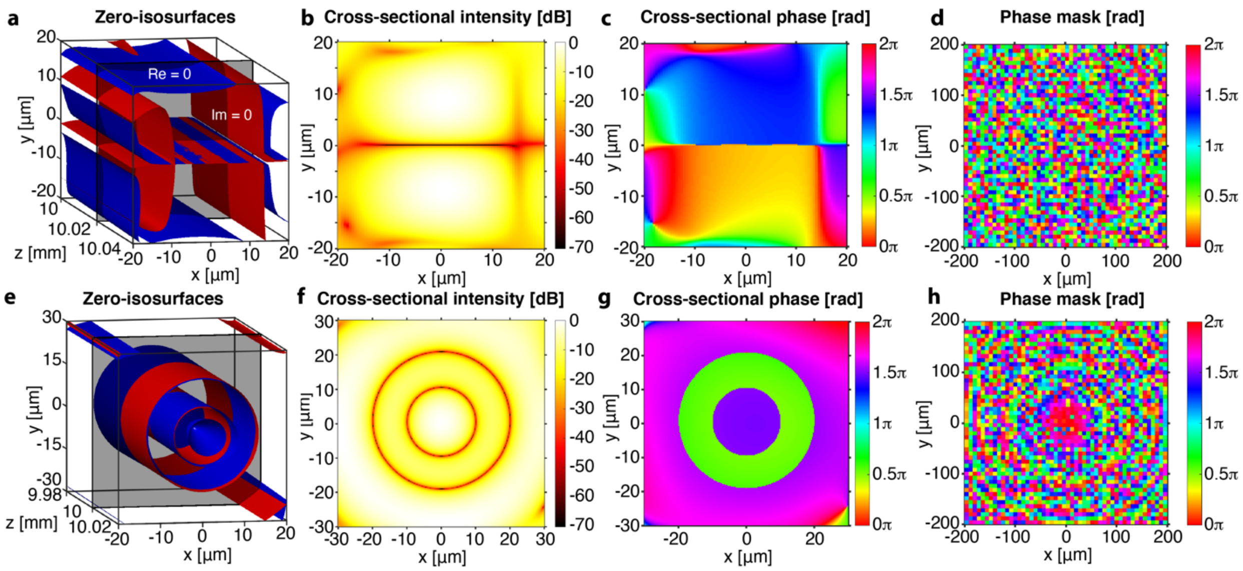

Supplementary Figure 6. **Additional phase singularity sheets engineered by phase gradient maximization.** Top row: a flat singularity sheet. Bottom row: a two-walled cylindrical singularity. **a** Real and imaginary field zero-isosurfaces for the flat sheet singularity. **b** Cross-sectional intensity and **c**, phase profiles at the grey z = 10.02 mm plane in **a**. **d** Phase mask placed at z = 0 mm which generates the flat singularity sheet structure. **e** Real and imaginary field zero-isosurfaces for the two-walled cylindrical sheet singularity. **f** Cross-sectional intensity and **g**, phase profiles at the grey z = 10 mm plane in **e**. **h** Phase mask placed at z = 0 mm which generates the two-walled cylindrical singularity sheet structure.

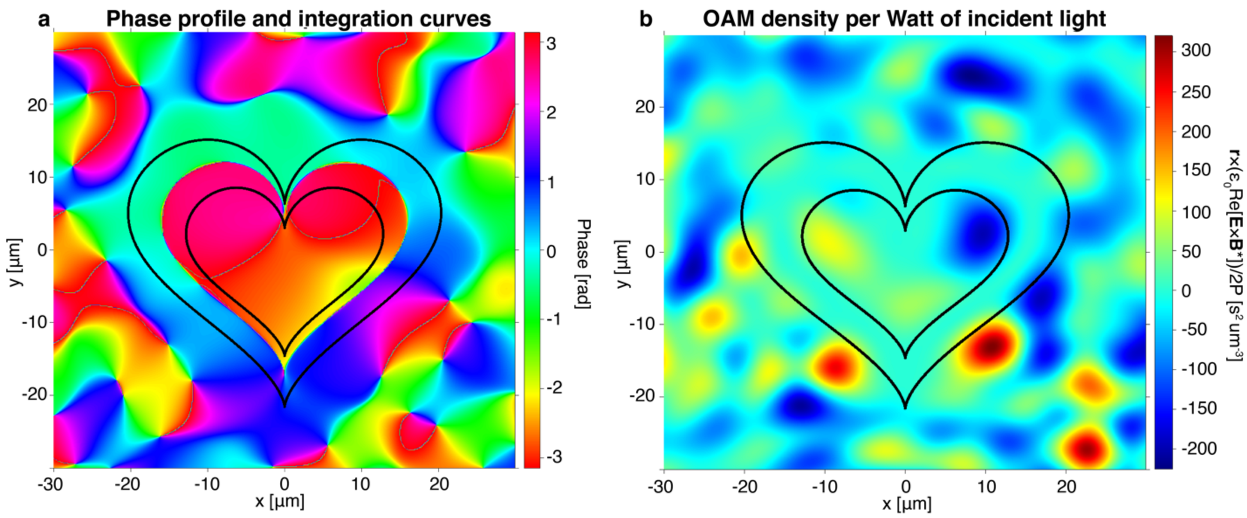

Supplementary Figure 7. **Contours used to compute the topological charge and mean orbital angular momentum (OAM) per photon. a** Inner and outer heart-shaped contours (black lines) used to compute the 2D heart-shaped phase sheet singularity topological charge by line integration. The surface plot shows the phase profile of the field at the z = 10 mm plane. The topological charge computed is $s = 0$. **b** Phase singularity sheet OAM density distribution and cross-sectional area (enclosed by the black lines) used to compute the mean OAM per photon l (in units of $\hbar$). The surface plot shows the time-averaged OAM density $\mathbf{r}\times\varepsilon_0\mathrm{Re}[\mathbf{E}\times\mathbf{B}^*])/2$ at the z = 10 mm plane, normalized to the incident power P passing through the metasurface aperture. The area integration for the time-averaged OAM and time-averaged energy per unit length is performed over the region between the same two heart-shaped contours as in **a** to yield l = -0.0011.

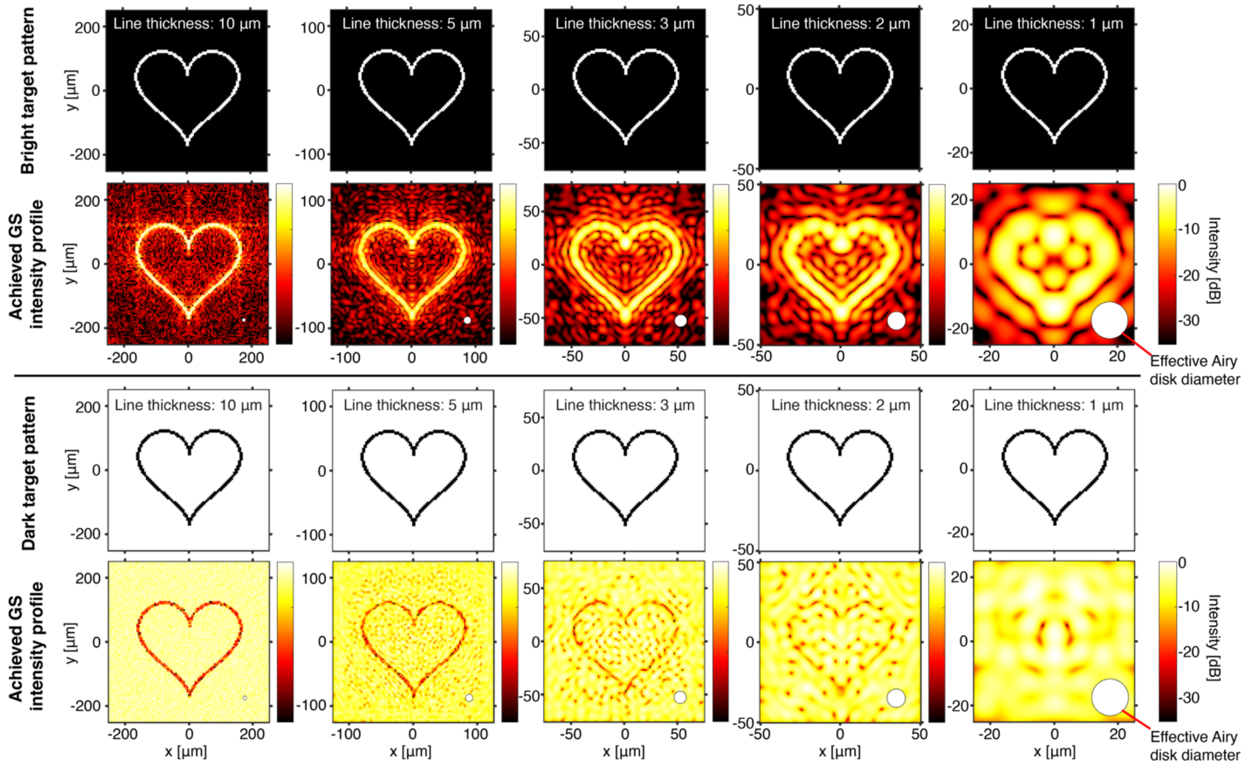

Supplementary Figure 8. **Results of Gerchberg-Saxton (GS) phase retrieval for designing bright and dark heart outlines using different line thicknesses.** The first two rows exhibit the results of using the GS algorithm for designing a bright heart shape against a dark background. The lower two rows exhibit the results of the same for designing a dark heart shape against a bright background. The geometry is the same as that used for the heart-shaped phase singularity sheet (aperture size 0.8 mm x 0.8 mm, 8 µm phase pixel pitch, 532 nm wavelength, 10 mm propagation distance). Target pattern linewidths decrease from left to right. The smallest, rightmost target heart pattern is identical in size to the heart-shaped singularity patterns studied in this paper. First row: the target pattern of a bright heart shape. Second row: achieved intensity plots (logarithmically scaled) for each target pattern, normalized to the maximum intensity achieved on that plane. The circle on the lower right corner shows the 11.5 µm diameter of the Airy disk minimum (for an aperture of diameter $\sqrt{2}\times 0.8$ mm) to exhibit the characteristic size of features on that plane. As the line thickness becomes much smaller than this characteristic size, the resultant intensity patterns become poorer replications of the ideal heart shape. Third row: target patterns of a dark heart shape. Fourth row: achieved intensity plots for each dark heart pattern, with the same 11.5 µm diameter Airy disk in the lower right corner for comparison. Again, pattern replication and contrast become poor as the line thickness is decreased.

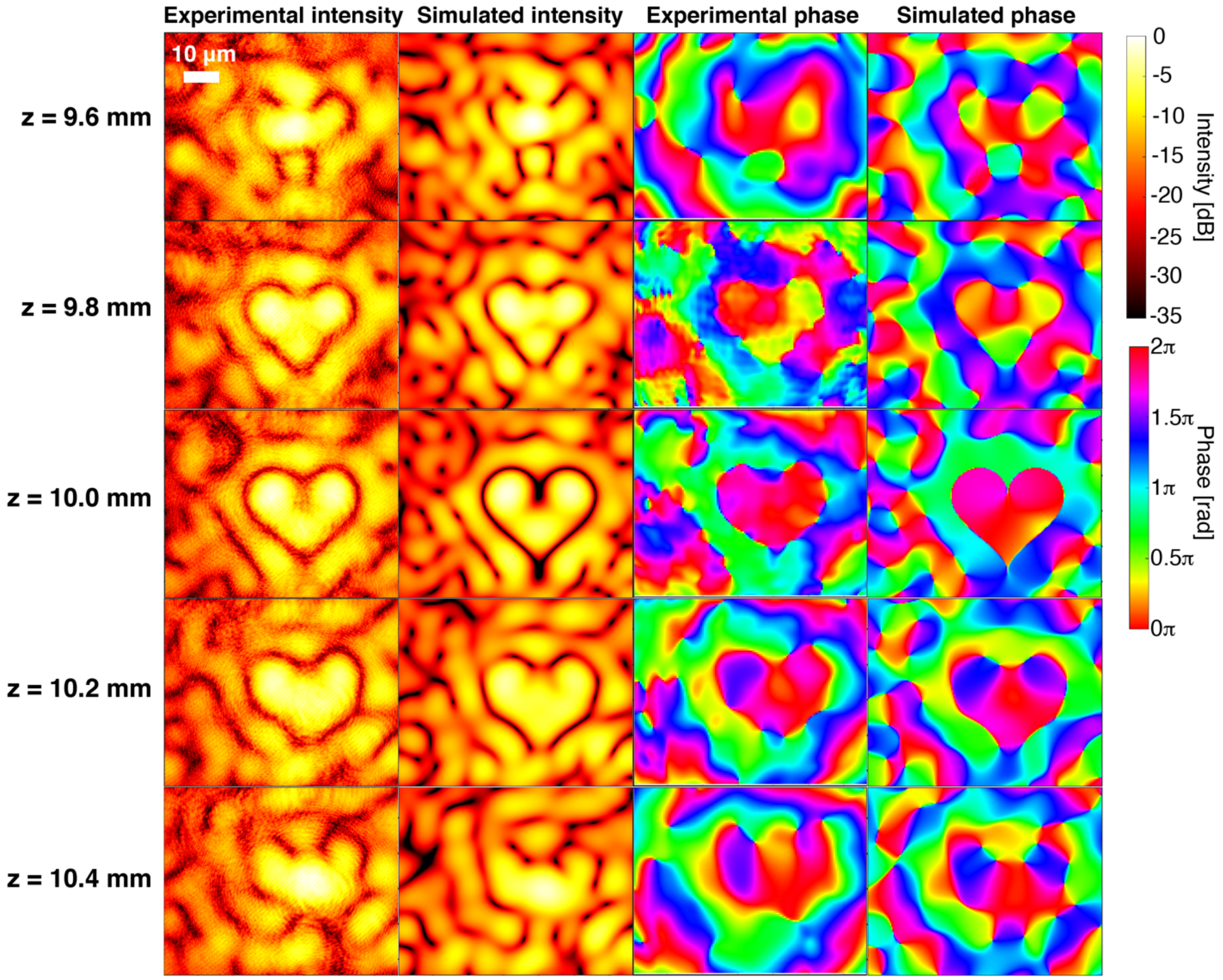

Supplementary Figure 9. **Cross-sections of the 2D heart-shaped phase singularity sheet at various transverse planes**. The columns are, from left to right, experimental intensity profile, simulated intensity profile, experimental retrieved phase profile, and the simulated phase profile. The sheet singularity breaks into a collection of 1D singularities away from the target plane of z = 10.0 mm.

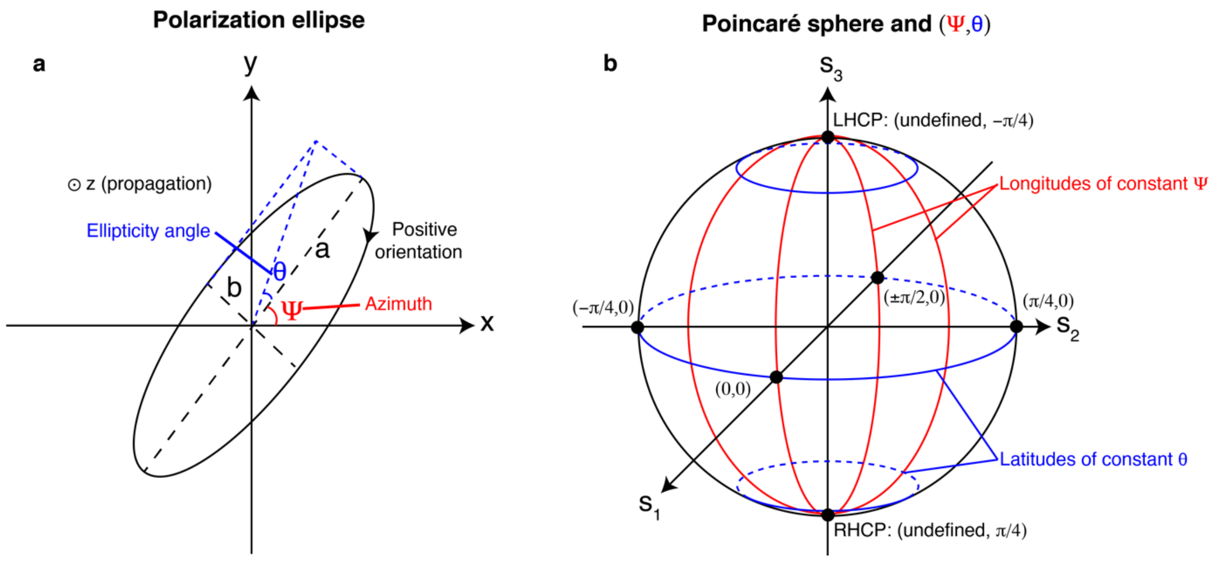

Supplementary Figure 10. **Polarization parameters and the Poincaré sphere. a** The electric field vector of a polarized beam propagating head-on in the *z*-direction traces out an ellipse in the *xy*-plane. This polarization ellipse can be parametrized by Ψ, the polarization azimuth, and θ=tan(b/a), the ellipticity angle. **b** Relationship between the Stokes parameters on the Poincaré sphere and (Ψ,θ). Isolines of Ψ are longitudes and isolines of θ are latitudes. The north and south poles of the sphere represent Left Handed Circular Polarization (LHCP) and Right Handed Circular Polarization (RHCP), respectively.

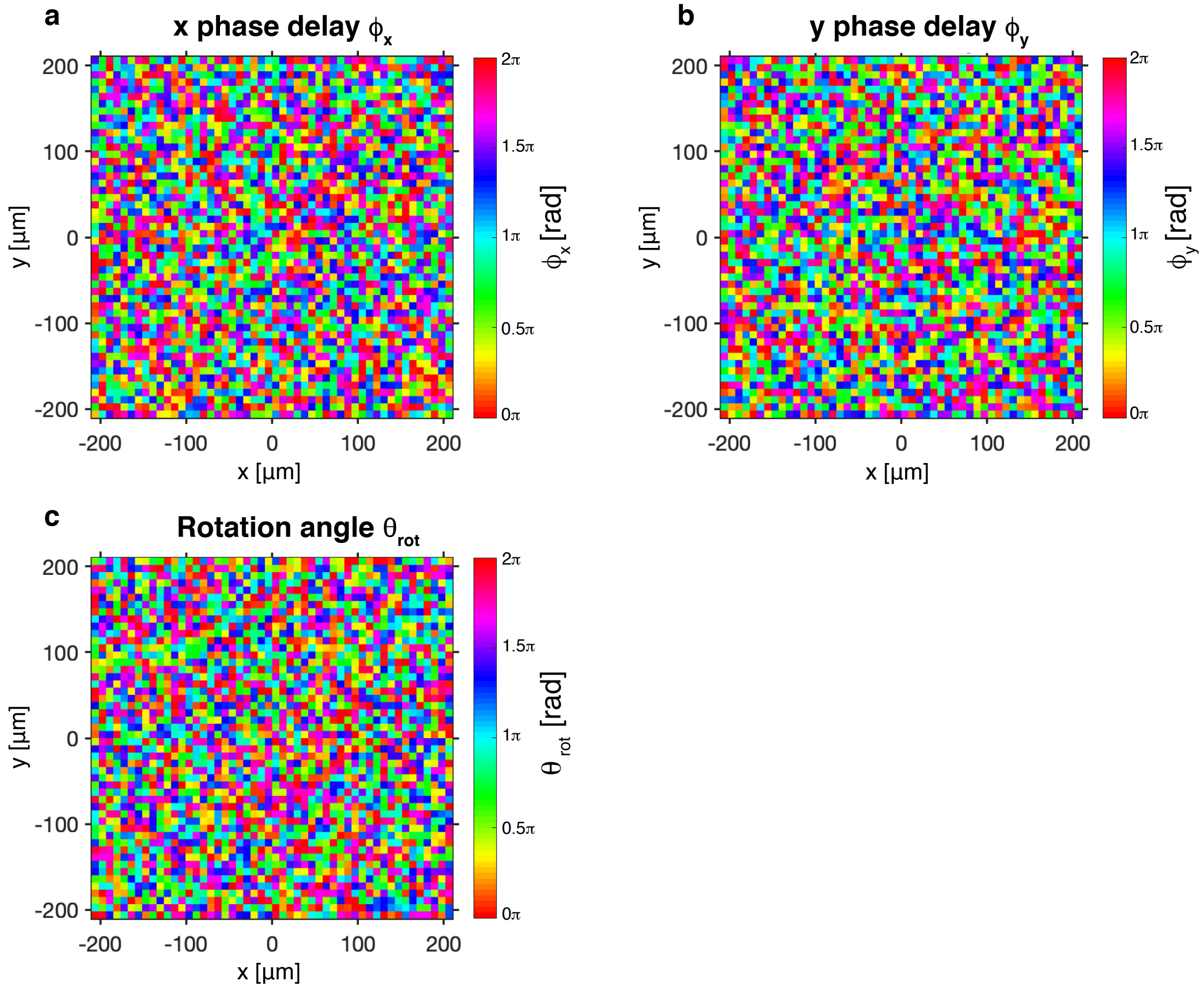

Supplementary Figure 11. **Optimized waveplate parameters for the heart-shaped 2D polarization singularity sheet. a** Required phase delay along the fast axis of the local waveplate φ_x. **b** Required phase delay in the transverse axis orthogonal to the fast axis of the local waveplate φ_y. **c** Required rotation angle of the fast axis of the local waveplate θ_{rot} relative to the laboratory *x*-direction.

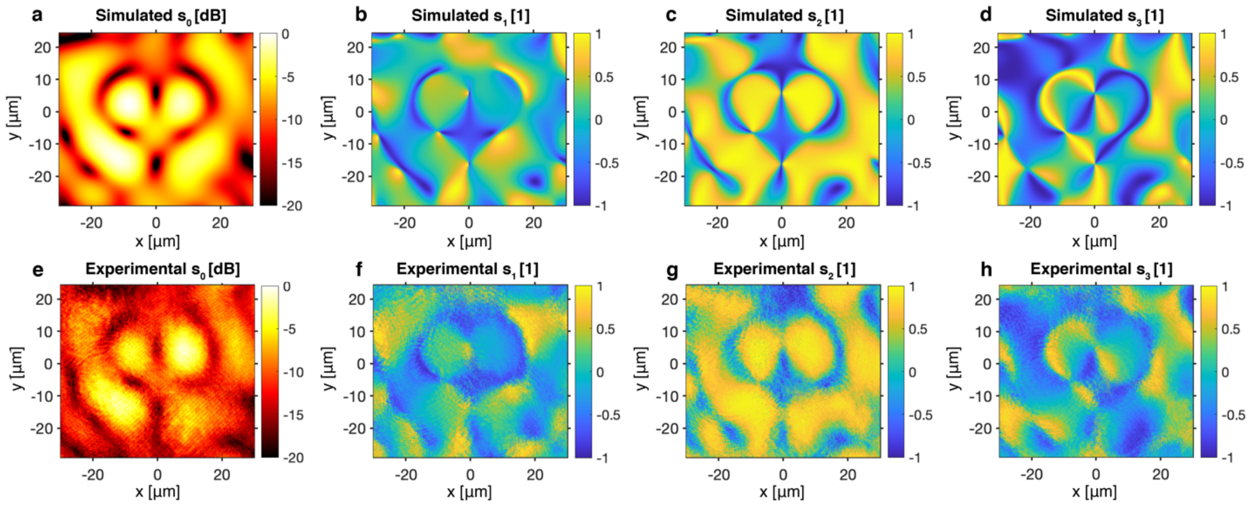

Supplementary Figure 12. **Comparison of the simulated (top row) and experimental (bottom row) Stokes parameters for the heart-shaped polarization singularity.** These are evaluated at the transverse plane z=10 mm. **a-d**, Simulated Stokes parameters s_0, s_1, s_2, and s_3 at the z=10 mm plane, respectively. s_0 is normalized to the maximum intensity on the plane and s_1, s_2, and s_3 are normalized to s_0. **e-h**, Experimentally measured Stokes parameters at the z=10 mm plane with the same normalization as in the simulated case.

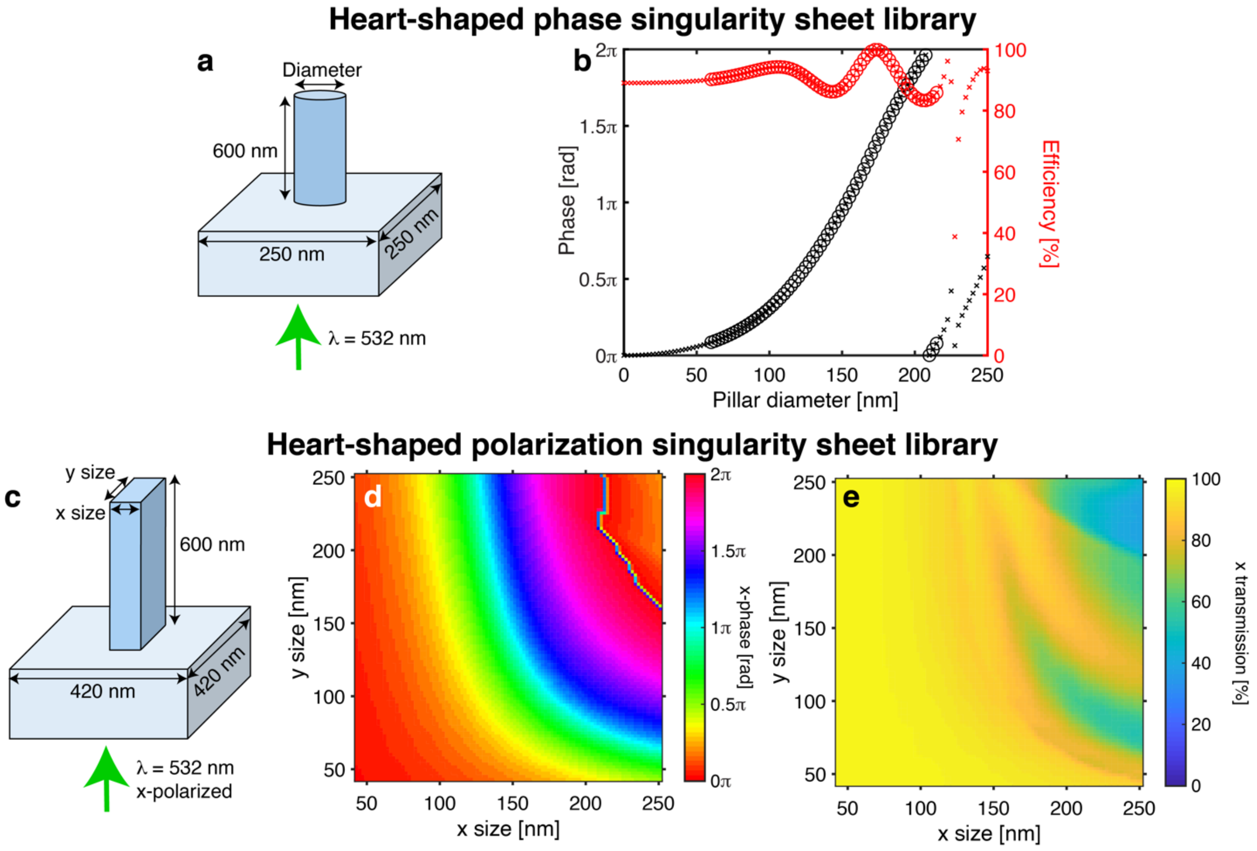

Supplementary Figure 13. **Meta-atom libraries for the experimental realization of metasurfaces that produce sheet singularity structures. a** Cylindrical meta-atom geometry used in realizing the heart-shaped phase singularity. **b** Transmission phase and efficiency dependence on the nanopillar diameter for the cylindrical meta-atom in **a**. The circled data points are used in the meta-atom library. The diameter range used is 60 nm to 215 nm, which provides 2π phase coverage. **c** Nanofin meta-atom geometry used in realizing the heart-shaped polarization singularity. **d** Phase of transmitted x-polarized light and **e**, efficiency in transmitting x-polarized light as a function of the x and y thicknesses of the nanofin.

SUPPLEMENTARY REFERENCES

1. Nocedal, J. & Wright, S. J. *Numerical Optimization*. (Springer, New York, 2006).

2. Weisstein, E. W. Heart Curve. *MathWorld* http://mathworld.wolfram.com/HeartCurve.html.

3. Hugonin, J. P. & Lalanne, P. Reticolo software for grating analysis. (2005).

4. Devlin, R. C., Khorasaninejad, M., Chen, W. T., Oh, J. & Capasso, F. Broadband high-efficiency dielectric metasurfaces for the visible spectrum. *Proceedings of the National Academy of Sciences of the United States of America* **113**, 10473–10478 (2016).

5. Khorasaninejad, M. *et al.* Metalenses at visible wavelengths: Diffraction-limited focusing and subwavelength resolution imaging. *Science* **352**, 1190–1194 (2016).

6. Balthasar Mueller, J. P., Rubin, N. A., Devlin, R. C., Groever, B. & Capasso, F. Metasurface Polarization Optics: Independent Phase Control of Arbitrary Orthogonal States of Polarization. *Physical Review Letters* **118**, 113901 (2017).

7. Pedrini, G., Osten, W. & Zhang, Y. Wave-front reconstruction from a sequence of interferograms recorded at different planes. *Optics Letters* **30**, 833-835 (2005).

8. Schaefer, B., Collett, E., Smyth, R., Barrett, D. & Fraher, B. Measuring the Stokes polarization parameters. *American Journal of Physics* **75**, 163–168 (2007).

9. Ostrovsky, A. S., Rickenstorff-Parrao, C. & Arrizón, V. Generation of the "perfect" optical vortex using a liquid-crystal spatial light modulator. *Optics Letters* **38**, 534–536 (2013)

10. Berry, M. Faster than Fourier. in *A Half-Century of Physical Asymptotics and Other Diversions* 483–493 (World Scientific, Singapore, 2017).

11. Sanjeev, A *et al*. Generation and Manipulation of Superoscillatory Hotspots Using Virtual Fourier Filtering and CTF Shaping. *Scientific Reports* **10**, 1-13 (2020)

12. Rogers, E.T.F *et al*. A super-oscillatory lens optical microscope for subwavelength imaging. *Nature Materials* **11**, 432-435 (2012).
